# Early Radiation Therapy Response Assessment Using Multi‐Scale Photoacoustic Imaging

**DOI:** 10.1002/advs.202509268

**Published:** 2026-03-09

**Authors:** Thierry L. Lefebvre, Mariam‐Eleni Oraiopoulou, Ellie V. Bunce, Thomas R. Else, Lorna C. Wright, Monika A. Golinska, Lina Hacker, Cara Brodie, Steven Kupczak, Yi Cheng, Lisa Young, Paul W. Sweeney, Sarah E. Bohndiek

**Affiliations:** ^1^ Department of Physics University of Cambridge Cambridge UK; ^2^ Cancer Research UK Cambridge Institute University of Cambridge Cambridge UK; ^3^ Department of Biostatistics and Translational Medicine Medical University of Lodz Lodz Poland; ^4^ Department of Oncology University of Oxford Oxford UK

**Keywords:** breast cancer, medical image analysis, photoacoustic imaging, radiation oncology, radiation therapy, treatment response

## Abstract

There is a critical unmet clinical need to identify biomarkers that predict and detect radiation therapy (RT) response in cancer. Using the unique capabilities of multi‐scale photoacoustic imaging (PAI) to depict tumor oxygenation and vasculature in vivo, we identified surrogate biomarkers of RT response in two human breast cancer xenograft models (MCF7 and MDA‐MB‐231), comparing hypofractionated delivery with an ablative single‐dose scheme. Mesoscopic and multispectral tomographic PAI were performed 24h pre‐RT, 24h post‐RT and at endpoint and were supported by ex vivo immunohistochemistry. MCF7 xenografts, which exhibited a denser and more mature vasculature, showed improved response to both RT schemes than MDA‐MB‐231, in terms of oxygenation, volume control and proliferation. Higher pre‐RT oxygenation and oxygen‐diffusion capacity were associated with improved outcome, consistent with the oxygen‐enhancement effect. PAI further revealed regimen‐specific vascular effects: ablative RT produced early pruning of looping vessels and superficial blood volume, while only hypofractionated RT led to a rise in intratumoral oxygenation at the endpoint in radiosensitive MCF7, indicative of reduced oxygen consumption in damaged tumor cells. We showed that PAI could capture early RT response and inform on radioresistance, thus demonstrating PAI as a promising tool to monitor the tumor vascular response to RT.

## Graphical abstract

Tomographic and mesoscopic photoacoustics capture intratumoural features of radioresistance and response. 

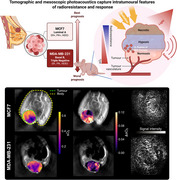

## 1 Introduction

Hypoxia is a common phenotype in many solid tumors. Uncontrolled cellular proliferation is metabolically demanding, yet the nascent tumor vasculature is often chaotic and poorly supported, leading to inadequate delivery and distribution of oxygen in the growing tumor mass [1]. Hypoxia and the associated overexpression of hypoxia‐related proteins is frequently associated with tumor aggressiveness, particularly in hormone‐dependent cancers such as breast cancer, and poor treatment outcomes, for example, in radiation therapy (RT) [2, 3, 4]. A particular use‐case that intersects these two challenges is the application of neoadjuvant RT in breast cancer treatment, which has shown promise for invasive and locally advanced breast cancer to enable improved resectability and breast‐conserving surgery [5, 6, 7, 8], and is currently being investigated in clinical trials (ClinicalTrials.gov ID, NCT05479409, NCT05216900, NCT06769919, NCT06313073, NCT06498154, and NCT05412225). In low lineal energy transfer (LET) RT such as X‐ray‐based therapies, the dose of radiation required to achieve the same biological effect in hypoxic regions is up to 3‐fold higher than in normoxic regions [9, 10]. Nonetheless, tumor oxygen distribution is typically not assessed or considered clinically during treatment planning, even with RT as a first‐line treatment in many solid tumors [11], let alone in the neoadjuvant setting.

With the trend toward hypofractionation, i.e., fewer fractions of higher dose using delivery methods such as stereotactic body/ablative RT (SBRT/SABR), hypoxia becomes a more critical consideration as the tumor microenvironment does not benefit as much from the inter‐fraction reoxygenation in conventional RT schemes [12, 13]. Real‐time visualization of the spatial distribution of intratumoral hypoxia is necessary to enable dose painting for hypofractionation schemes [14]. Furthermore, in ablative courses of RT prescribed with higher dose per fraction (>16 Gy), endothelial cells lining the vasculature will undergo apoptosis and contribute to the tumor response [15, 16, 17]. Damage to endothelial cells can be mediated through acid sphingomyelinase‐dependent and p53‐independant membrane alterations, leading to ceramide upregulation and apoptotic signaling, which could acutely exacerbate hypoxia, worsening outcomes specifically through the vascular response to SBRT/SABR [18, 19, 20].

The clinical value of imaging in the neoadjuvant RT setting is well established for predicting and assessing treatment response [21, 22]; however, it has yet to account for the impact of hypoxia [14, 23, 24]. Positron emission tomography (PET) tracers like fluoromisonidazole (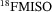) could be used to map tumor hypoxia [25, 26, 27], but adding radiation dose is undesirable for longitudinal imaging. Magnetic resonance imaging (MRI) can determine microcirculatory properties through blood‐ or tissue‐oxygen level dependent signals [28, 29], define sub‐volumes for radiation dose escalation based on diffusion [30, 31] and identify perfusion imaging biomarkers [32, 33]. Nonetheless, these techniques are constrained by limited spatio‐temporal resolution and often require the use of exogenous contrast agents, which comes with costs and potential toxicity concerns [34, 35]. Considering the spatio‐temporal heterogeneity of hypoxia and angiogenesis in the tumor microenvironment [1, 36, 37], any solution applied in the neoadjuvant setting would need to be low‐cost, easy to use at the bedside, and provide high‐resolution data for immediate interpretation.

In this study, we demonstrate the potential of photoacoustic imaging (PAI) as a solution to these key challenges in radiation oncology [38, 39], demonstrating its value in providing predictive biomarkers, enabling early response monitoring, and visualization of vascular remodeling. PAI leverages optical contrast for mapping different tissue chromophores at depth by overcoming the optical diffusion limit through ultrasound detection of acoustic relaxation waves. The characteristic high optical absorption contrast of oxy‐ and deoxy‐haemoglobin is ideally suited for visualizing perfused vasculature and quantifying tumor blood oxygenation [40, 41]. PAI can be applied tomographically at high temporal resolution or at higher spatial resolution with raster‐scanning modes [42, 43]. Previous studies have shown the ability of PAI to capture RT and chemo‐RT response in preclinical models separately using these distinct PAI modes [44, 45, 46]. However, each examined only part of the roles of the vasculature and tumor oxygenation in the RT response picture. Here, we holistically combined tomographic and mesoscopic PAI, bringing together their respective insights in tumor vascular function and morphology. We undertook a longitudinal investigation to establish the potential of the derived quantitative PAI biomarkers in two preclinical breast cancer models at 24h before RT treatment, 24h after, and at endpoint (∼1 week after) with hypofractionated and ablative schemes. Using a thorough immunohistochemistry (IHC) analysis of the models, we demonstrate that PAI captures changes in vascular morphology, including vessel looping structures, alongside intratumoral oxygenation dynamics, which together predict response to treatment, define the early response to treatment and confirming response at endpoint. PAI could therefore play a future role in profiling radiosensitivity, treatment planning and verification, as well as confirming treatment response in the neoadjuvant and adjuvant settings.

## 2 Results

**FIGURE 1:**
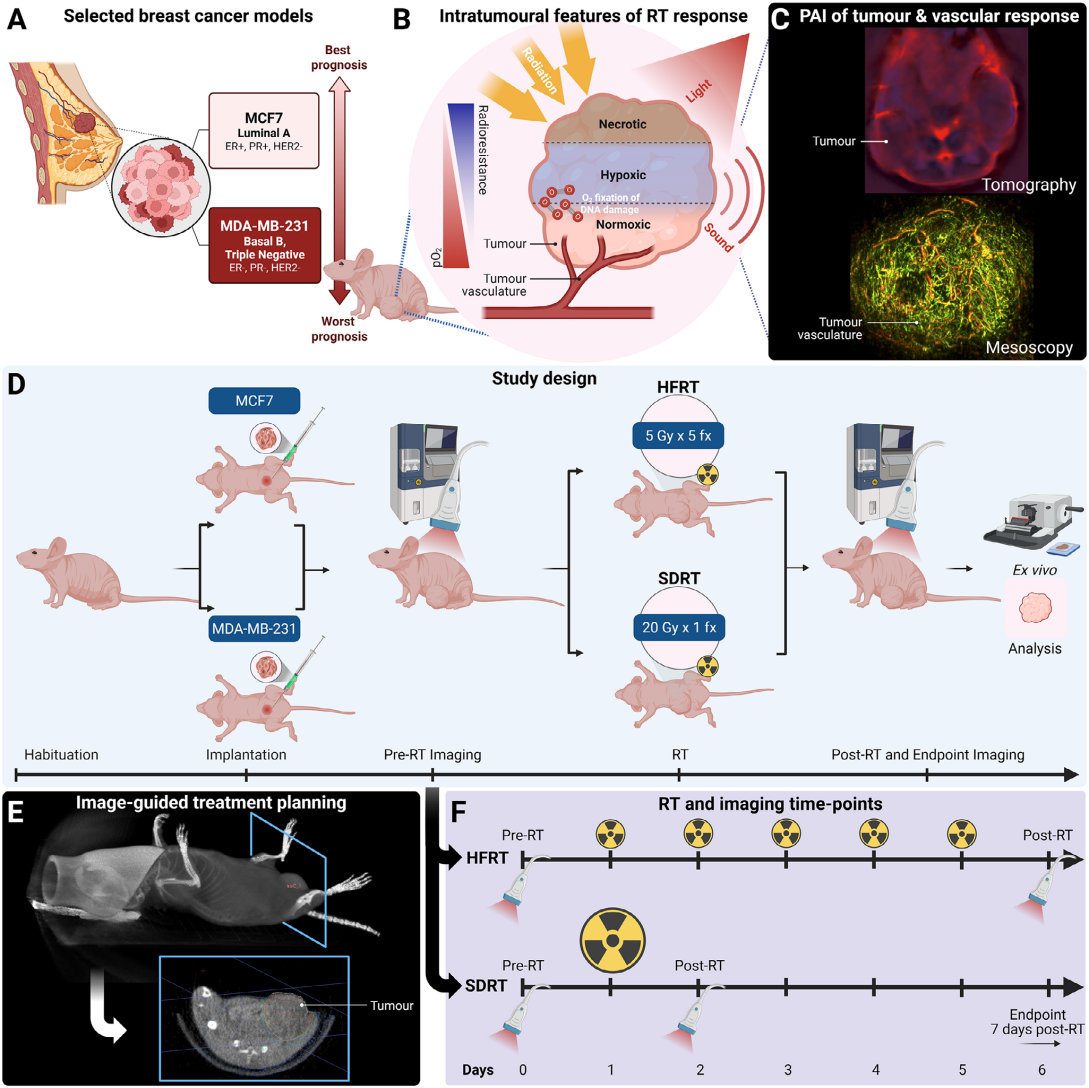
Study Design. (A) The human cell lines MCF7, a luminal A subtype ER+ and PR+ breast cancer model, and MDA‐MB‐231, a triple negative basal B subtype breast cancer model, were selected based on their differences in aggressiveness and radioresistance [47, 48, 49, 50]. (B) Intratumoral features of cancer, such as hypoxia, induce radiation therapy resistance, and the vasculature, being the principal input of molecular oxygen, plays a key role in tumor oxygenation. (C) Tumor vasculature can be imaged using photoacoustic imaging, in a tomographic configuration for functional analysis of oxygenation (upper panel), or in a planar raster‐scanning configuration at the meso‐scale for morphological vascular characterization (lower panel). (D) Study timeline with breast cancer models enrolled into either the control group, or one of the treated groups, i.e., hypofractionated radiation therapy (HFRT) with 5 Gy per fraction delivered in 5 fractions, or single dose radiation therapy (SDRT) with 20 Gy delivered in a single fraction. (E) Treatment simulation on cone‐beam computed tomography with a single beam delivered in a moving arc. (F) Timeline of imaging and radiation delivery in each treated group, with imaging conducted 24h before, 24h after radiation therapy, and at endpoint (7 days following treatment on average). Created with Biorender.com.

Two human breast cancer xenograft models (oestrogen‐dependent MCF7 and triple negative MDA‐MB‐231) were selected for their established differences in aggressiveness and radioresistance (Figure 1A) [47, 48, 49, 50]. Engrafted mice were enrolled in the study once tumors in the position of the mammary fat pad reached ∼400 mm^3^ in volume and were imaged at baseline and longitudinally with in vivo photoacoustic imaging (PAI) to capture intratumoral features of radiation therapy (RT) response (Figure 1B,C). Enrolled mice were randomized into control, hypofractionated RT (HFRT; 5×5 Gy) or single‐dose RT (SDRT; 1×20 Gy) groups, and computed tomographic image‐guided treatments were delivered with dynamic arcs using the small animal radiation research platform (SARRP) mirroring clinical standards (Figure 1D–F) [51]. Change in tumor volume monitored by calliper measurements informed treatment response and was complemented by ex vivo immunohistochemistry (IHC)‐based analysis of tumor cross‐sections excised and processed at endpoint (∼7 days post‐RT on average).

### 2.1 Oestrogen‐Dependent MCF7 Tumors Demonstrate Greater Radiosensitivity Than Triple Negative MDA‐MB‐231 Tumors, With Improved Outcomes Using a Hypofractionated Scheme

**FIGURE 2:**
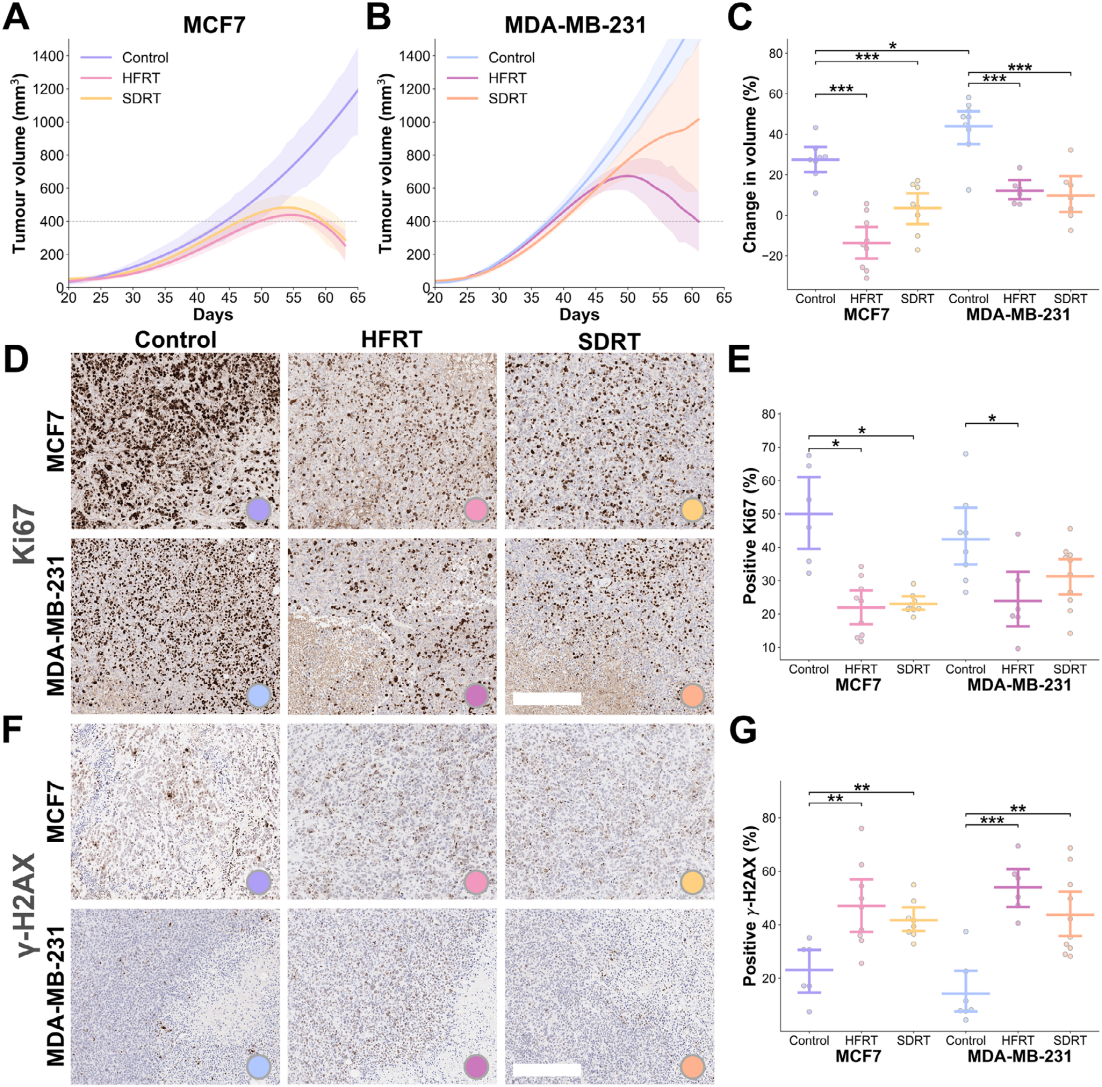
Evaluation of radiation therapy response in the preclinical breast cancer models. Differences in response to selected radiation therapy schemes in the two breast cancer xenografts were assessed with tumor volume and ex vivo immunohistochemistry in resected tumors at endpoint. Growth curves assessed by calliper measurements in (A) MCF7 (*n* = 23) and (B) MDA‐MB‐231 (*n* = 23) models for the untreated (Control; *n* = 13), hypofractionated (HFRT)‐treated (*n* = 15), and single dose (SDRT)‐treated (*n* = 18) groups. Full lines represent average group‐wise spline‐fitted growth curves, shaded areas are 90% confidence intervals, and dashed lines represent the average enrolment size of 400 mm^3^. (C) Percent change in measured tumor volume between enrolment and endpoint (7 days on average post‐radiation therapy) demonstrating growth inhibition in all treatment groups. Ex vivo immunohistochemistry sections for (D) proliferation on Ki67, quantified in (E) bar and point plots, and (F) DNA damage on γ‐H2AX, quantified in (G) bar and point plots, with bars representing the mean and 90% confidence intervals. Scale bar, 200 μm. *P*‐values are calculated using two‐tailed Mann–Whitney *U*‐test with Holm–Bonferroni correction: *P < 0.05, **P < 0.01, ***P < 0.001.

We first sought to profile the model‐specific radiosensitivity and response to the investigated RT regimens, based on tumor volume and IHC markers of proliferation and DNA damage. As would be expected from the more aggressive triple negative breast cancer model, MDA‐MB‐231 tumors reached the enrolment volume of 400 mm^3^ faster than MCF7 (39.3 ± 6.4 days vs. 47.0 ± 8.2 days, *P* =0.0058, Figure 2A,B and Figure S1) and when left untreated, showed a greater volume increase from enrolment to endpoint compared to MCF7 xenografts (change in volume, 44.01% ± 13.60% vs. 27.46% ± 9.29%, *P* =0.0104; Figure 2C). Following RT, all tumors demonstrated growth inhibition compared to controls across models and RT schemes (*P*<0.001 for all). The largest change in tumor volume between enrolment and endpoint was observed in HFRT‐treated MCF7 compared to controls (Figure 2C; –13.65% ± 13.15% vs. 27.46% ± 9.29%, *P* <0.0001), followed by SDRT‐treated MCF7 xenografts (3.59% ± 12.27%, *P* = 0.0006), HFRT‐treated MDA‐MB‐231 compared to untreated controls (12.20% ± 6.66% vs. 44.01% ± 13.60%, *P* =0.00018), and SDRT‐treated MDA‐MB‐231 (9.70% ± 12.97%, *P* = 0.00036).

The most responsive groups also displayed decreased proliferation, as shown by Ki67 on IHC, with significantly lower percentage of Ki67 positive nuclei in HFRT‐treated MCF7 xenografts compared to controls (Figure 2D,E; 21.98% ± 8.37% vs. 50.04% ± 14.60%, *P* =0.011), and in the SDRT‐treated MCF7 group (23.09% ± 3.08%, *P* = 0.012). Proliferation in MDA‐MB‐231 tumors was significantly lower only in the HFRT arm (42.45% ± 13.32% vs. 23.95% ± 11.78%, *P* =0.019). Similarly, DNA damage measured with γ‐H2AX IHC was significantly increased in all treated groups compared to control mice, across tumor models (Figure 2F,G; *P*<0.01 for all). The measured change in tumor volume was correlated with ex vivo IHC markers of response, Ki67 and γ‐H2AX (*r* = 0.66, *P* = 0.0030; *r* = –0.72, *P* = 0.0008. respectively). Necrotic areas were higher in treated groups than in controls in both models (*P*<0.05, except for SDRT‐treated MCF7 vs. MCF7 controls; Figure S2). Thus, the oestrogen‐dependant MCF7 model was confirmed to be a more radiosensitive breast cancer model than the MDA‐MB‐231 triple negative breast cancer model. The hypofractionated scheme (HFRT) resulted in improved tumor control in both models, compared to the stereotactic ablative scheme (SDRT), highlighting the benefits of fractionation.

**FIGURE 3:**
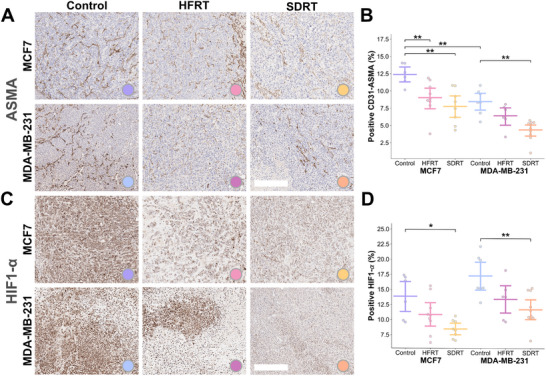
Differences in tumor vasculature and hypoxia after radiation therapy in breast cancer xenografts assessed with ex vivo immunohistochemistry. Ex vivo immunohistochemistry sections quantified for (A) double CD31‐ASMA representing mature vasculature coverage (with ASMA sections displayed) in (B) bar and point plots, and (C) hypoxia on HIF1‐α in (D) bar and point plots, with bars representing mean and 90% confidence intervals. Note that colors are used consistently throughout the manuscript, with: warm color tones representing SDRT, respectively in yellow and orange for MCF7 and MDA‐MB‐231; mid tones being used for HFRT, respectively in pink and magenta; and cool tones are used for controls, respectively in purple and blue. Scale bar, 200 μm. *P*‐values are calculated using two‐tailed Mann–Whitney *U*‐test with Holm–Bonferroni correction: *P < 0.05, **P < 0.01, ***P < 0.001.

To assess the tumor vascular microenvironment in both models, blood vasculature and intratumoral hypoxia were also characterized at endpoint on IHC. Double positive areas on serial sections of CD31, capturing endothelial cells, and ASMA, capturing smooth muscle cells, and taken together to reflect vascular maturity, were higher in MCF7 than in the MDA‐MB‐231 tumor sections in the control group (percent CD31‐ASMA positive areas, 12.39% ± 1.52% vs. 8.46% ± 1.83%, *P* =0.0014; Figure 3A,B). The HIF1‐α positive area, taken to reflect hypoxia, was also lower in MCF7 xenografts than in MDA‐MB‐231, but not significantly (13.13% ± 4.83% vs. 17.28% ± 3.44%, *P* =0.11; Figure 3C,D). When assessing treated groups, RT‐treated tumors displayed significantly less mature vasculature as seen on CD31‐ASMA, compared to controls in both models (*P* < 0.01, except HFRT‐treated MDA‐MB‐321 tumors). HIF1‐α staining was only significantly decreased in SDRT‐treated tumor sections compared to controls in both models (MCF7, *P* = 0.047; and MDA‐MB‐231, *P* = 0.0045). Hence, MCF7 xenografts were confirmed to possess improved vessel coverage and intratumoral oxygenation in comparison to MDA‐MB‐231 xenografts, and RT was shown to alter the tumor vascular microenvironment, with greater reduction in vessel maturity and HIF1‐α staining observed in SDRT‐treated tumors.

### 2.2 Photoacoustic Imaging Reveals Clear Differences in Vascular Microenvironments at Baseline in the Two Models That Correlate With Radiation Therapy Outcomes

We next sought to assess model‐specific PAI‐detectable in vivo intratumoral features prior to the administration of any treatment to inform on radiosensitivity. Multi‐scale PAI comprised multispectral tomographic acquisitions to quantify total haemoglobin (THb) and blood oxygen saturation (${\mathrm{sO}}_{2}$), including an air‐to‐oxygen breathing challenge to derive ΔsO 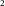 and responding fraction (RF) [42, 52, 53], and high‐resolution mesoscopic raster scans to characterise peripheral vascular morphology, captured within the first 4 mm from tissue surface, and quantified following blood vessel segmentation [54] to extract total blood volume (BV), vessel diameters, perfused vascular density, and the count of looping structures.

**FIGURE 4:**
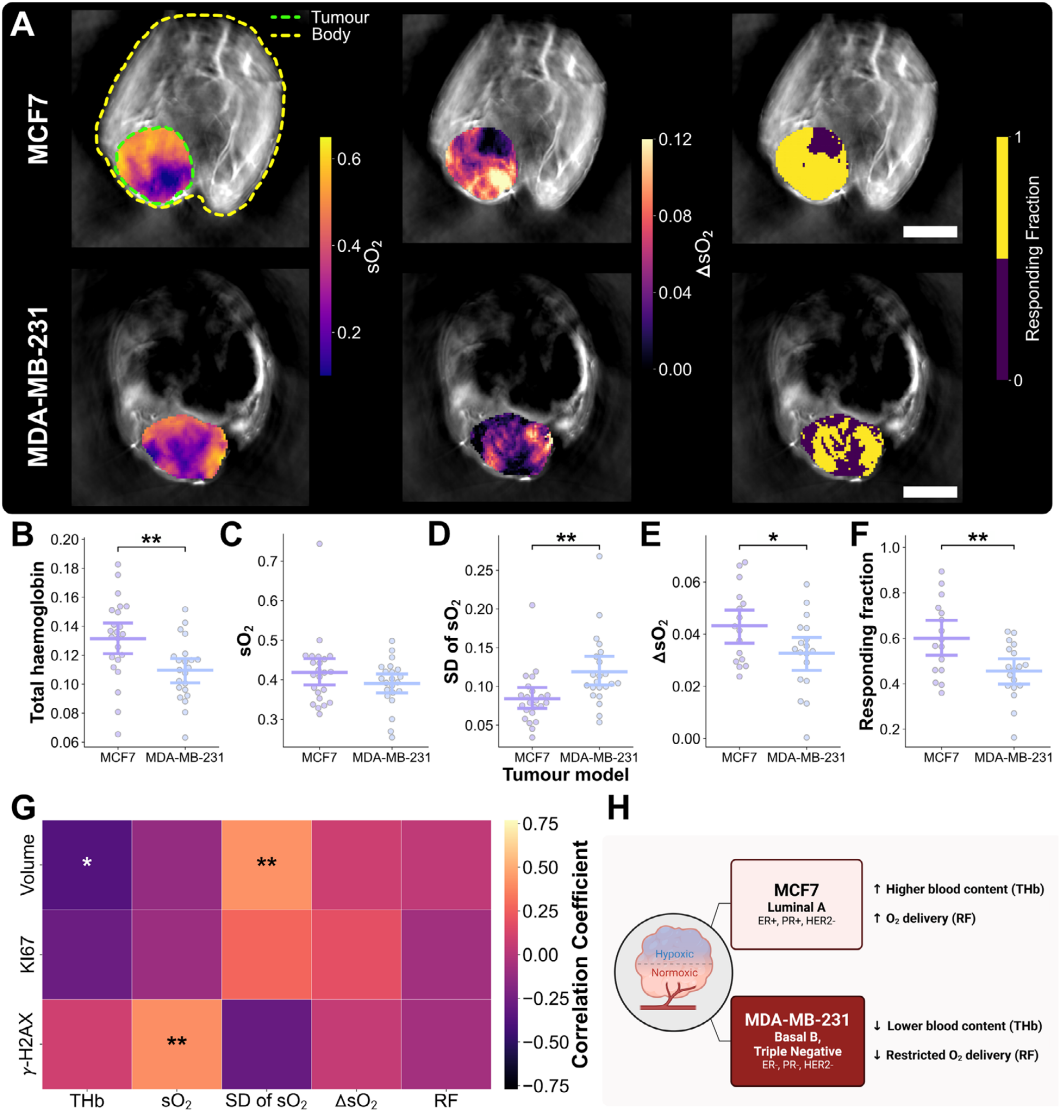
Tomographic PAI delineates models based on intratumoral blood content and oxygenation at baseline. (A) Exemplar MCF7 and MDA‐MB‐231 tumor‐bearing mice imaged at baseline (enrolment) with multispectral tomographic PAI, overlaid with quantitative parametric maps (from left to right) of ${\mathrm{sO}}_{2}$, and in response to a breathing gas challenge, ΔsO 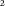 and responding fraction. Scale bars, 2 mm. Bar plots of (B) total haemoglobin, (C) blood oxygen saturation (${\mathrm{sO}}_{2}$) (D) spatial standard deviation (SD) of ${\mathrm{sO}}_{2}$, (E) ΔsO 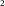 under gas challenge, and (F) responding fraction where each point represents an independent tumor in MCF7 (*n* = 23) or MDA‐MB‐231 group (*n* = 23), with bars representing mean and 90% confidence intervals. (G) Correlation heatmap of in vivo tomographic PAI quantitative biomarkers and ex vivo immunohistochemistry parameters. (H) Summary schematic of vascular microenvironment assessment using baseline tomographic PAI biomarkers in both models. *P*‐values are calculated using two‐tailed Mann–Whitney *U*‐test with Holm–Bonferroni correction: *P < 0.05, **P < 0.01, ***P < 0.001. Created with BioRender.com.

At enrolment, MCF7 and MDA‐MB‐231 xenografts were imaged with tomographic PAI 24h pre‐RT (Figure 4A). Prior to the administration of any treatment, THb, taken to reflect intratumoral blood content, was higher in MCF7 tumors than MDA‐MB‐231 tumors (0.126 ± 0.026 vs. 0.102 ± 0.020, *P* =0.0032; Figure 4B), as was ${\mathrm{sO}}_{2}$, a proxy of vascular blood oxygenation, although non‐significantly (0.408 ± 0.096 vs. 0.362 ± 0.055, *P* =0.050; Figure 4C), in line with previous findings in these models [55]. Intratumoral distribution of ${\mathrm{sO}}_{2}$ displayed less heterogeneity in the MCF7 compared to the MDA‐MB‐231 model (SD of ${\mathrm{sO}}_{2}$ pre‐RT, 0.091 ± 0.035 vs. 0.137 ± 0.050, *P* =0.047; Figure 4D). Upon administration of a change in breathing gas to 100% oxygen, the change in ${\mathrm{sO}}_{2}$ and the fraction of pixels responding to gas challenge were both higher in the MCF7 tumors compared to the MDA‐MB‐231 (ΔsO 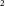 pre‐RT, 0.043 ± 0.014 vs. 0.034 ± 0.013, *P* =0.039; RF pre‐RT, 0.588 ± 0.188 vs. 0.469 ± 0.097, *P* =0.018 Figure 4E,F) indicating greater vascular maturity and the ability for oxygen to diffuse across tumor vasculature.

We evaluated correlations across both models between pre‐RT PAI biomarkers and endpoint histopathological IHC markers to assess the potential of tomographic PAI to reflect the underlying molecular changes that could predict future RT response. THb and SD of ${\mathrm{sO}}_{2}$ at baseline were correlated with the change in tumor volume (*r* = ‐0.38, *P* = 0.016, and *r* = 0.42, *P* = 0.0041, respectively; Figure 4G). Baseline ${\mathrm{sO}}_{2}$ also showed a significant correlation with γ‐H2AX (*r* = 0.41, *P* = 0.0059; Figure 4G). These findings indicate that increased blood content pre‐RT and lower heterogeneity in oxygenation were associated with improved RT efficacy. Higher tissue oxygenation was related to a higher extent of DNA damage, which would be expected given the well‐known oxygen enhancement effect. Overall, using tomographic PAI, MDA‐MB‐231 xenograft tumors displayed higher tissue oxygenation heterogeneity, lower tumor blood content, and weaker intratumoral oxygen diffusion ability than the MCF7 model, which correlated with poorer treatment response at endpoint (Figure 4H).

**FIGURE 5:**
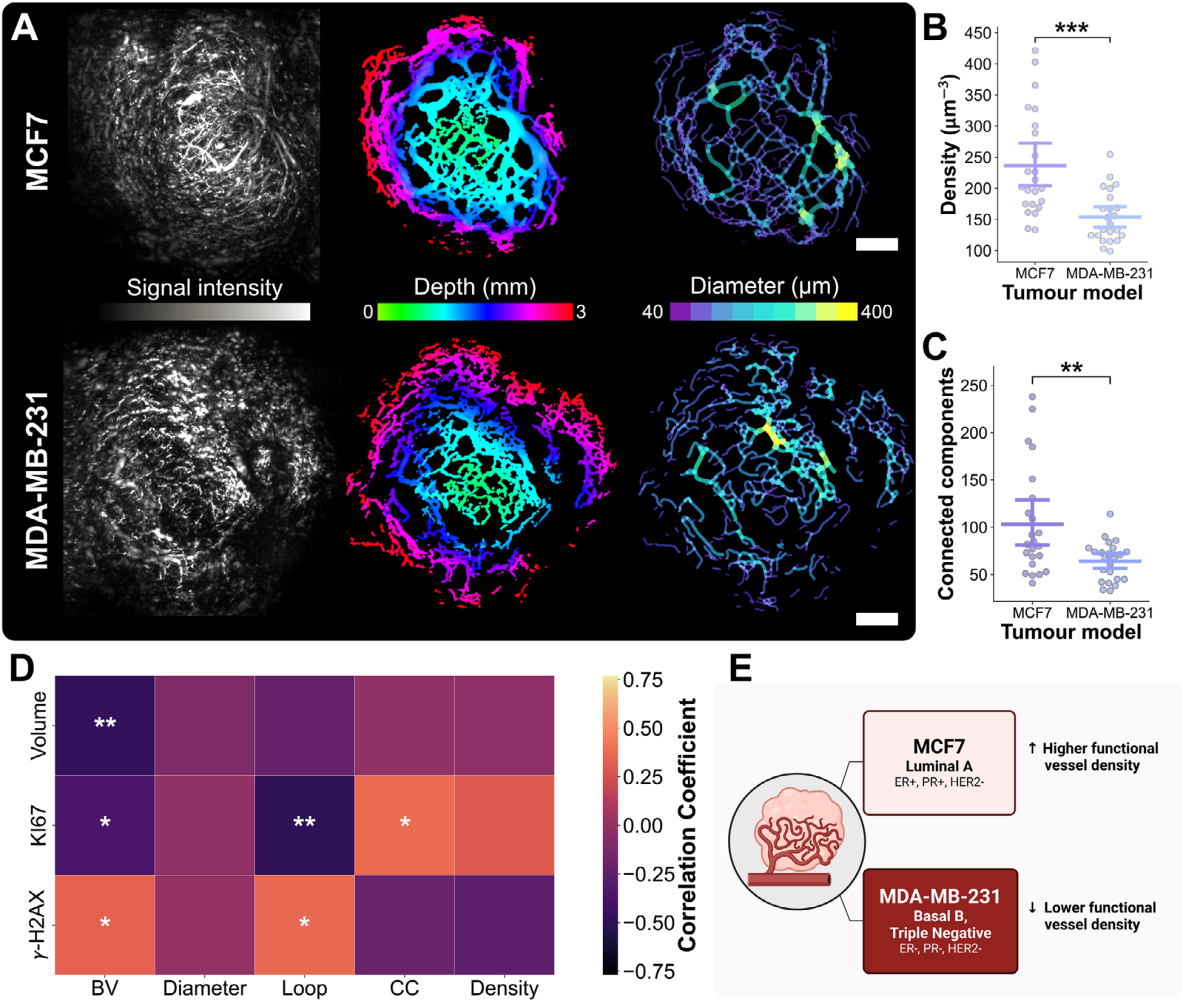
Mesoscopic PAI detects underpinning differences in vascular density and architecture. (A) MCF7 and MDA‐MB‐231 tumor‐bearing mice imaged at baseline (enrolment) showing (from left to right) mesoscopic PAI signal intensity, depth‐resolved segmented vasculature and a skeletonized diameter map. Scale bars, 2 mm. Bar plots of (B) mesoscopic PAI vascular density and (C) number of connected components across the vascular networks in MCF7 (*n* = 23) or MDA‐MB‐231 group (*n* = 23) with bars representing mean and 90% confidence intervals. (D) Correlation heatmap of in vivo mesoscopic PAI quantitative biomarkers and ex vivo immunohistochemistry parameters. (E) Summary schematic of vascular microenvironment assessment using baseline mesoscopic PAI biomarkers in both models. *P*‐values are calculated using two‐tailed Mann–Whitney *U*‐test with Holm–Bonferroni correction: *P < 0.05, **P < 0.01, ***P < 0.001. Created with BioRender.com.

Considering the vascular architecture in more detail, mesoscopic PAI indicated a denser peripheral perfused vasculature in MCF7 xenografts compared to MDA‐MB‐231 xenografts (vascular density, 236.4 ± 84.5 μm 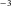 vs. 153.9 ± 41.2 μm 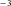, *P* =0.00006; Figure 5A,B). A higher number of vascular connected components were also observed in MCF7 tumors, corresponding to the number of perfused vessel segments connected in the captured vascular skeletons (103.13 ± 57.48 vs. 64.09 ± 20.61, *P* = 0.00185; Figure 5C). Although the density of vascular networks between models was different, the overall vascular morphology (blood volume, diameters and looping structures, Figure S3) captured close to the tumor surface was not significantly different between MCF7 and MDA‐MB‐231 tumors at baseline. The measured pre‐RT blood volume (BV) was significantly correlated with biomarkers of RT response at endpoint (change in tumor volume *r* = –0.34, *P* = 0.022, Ki67 *r* = –0.48, *P* = 0.0016, γ‐H2AX *r* = 0.50, *P* = 0.0017; Figure 5D). Similarly, increased loops normalized to BV was associated with decreased proliferation (Ki67 *r* = ‐0.49, *P* = 0.0018) and increased DNA damage (γ‐H2AX *r* = 0.36, *P* = 0.034). Overall, MCF7 xenografts displayed a denser perfused vascular network at the tumor periphery compared to that of the MDA‐MB‐231, and measured BV and looping structures were associated with improved tumor response to RT (Figure 5E).

### 2.3 Longitudinal Photoacoustic Imaging can Predict and Detect Early Tumor Response to Radiation Therapy

To track functional and morphological vascular changes after irradiation, tomographic and mesoscopic PAI biomarkers were reassessed at 24h post‐RT and at endpoint and expressed relative to pre‐RT baseline.

**FIGURE 6:**
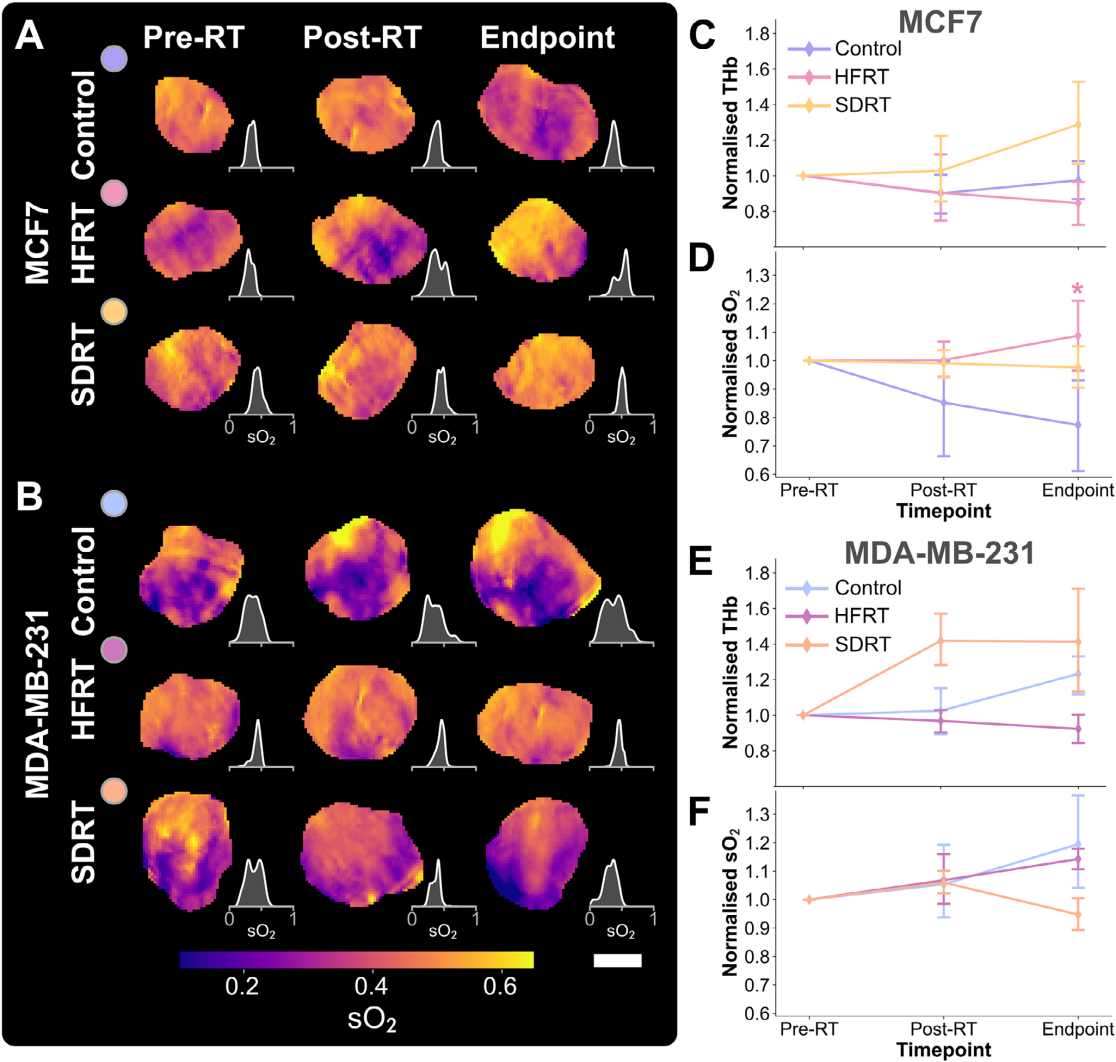
Longitudinal in vivo tomographic photoacoustic imaging detects radiation therapy response in breast cancer models. Exemplar blood oxygen saturation tumor maps with paired density plots in (A) MCF7 and (B) MDA‐MB‐231 xenografts, across time‐points and treatment groups. Scale bar, 2 mm. Tomographic photoacoustic imaging biomarkers normalized to pre‐treatment scan in MCF7 and MDA‐MB‐231 tumor‐bearing mice, respectively, (C,D) total haemoglobin (THb), and (E,F) blood oxygen saturation (sO_2_). All imaging biomarkers in panels (C‐F) are normalized to 1 at the pre‐RT time‐point. Point and bars represent mean and standard deviation in MCF7 groups (control *n* = 6, HFRT *n* = 9, or SDRT *n* = 8) and MDA‐MB‐231 groups (control *n* = 7, HFRT *n* = 6, or SDRT *n* = 10). Exact endpoint days are provided in Table S1. *P*‐values are calculated using two‐tailed Mann–Whitney *U*‐test with Holm–Bonferroni correction: *P < 0.05, **P < 0.01, ***P < 0.001.

First, PAI tomography scans acquired longitudinally, including at 24h post‐RT in both treatment arms were quantified (Figure 6A,B). On tomographic PAI, the change in THb from baseline across time‐points, tumor models and treatment arms was explained with linear mixed effects (LME) modeling. On average, THb increased over time‐points, in groups treated with SDRT, and differed between models (time‐point normalized regression coefficient (NRC), 0.069 ± 0.026, *P* = 0.009; SDRT arm NRC, 0.159 ± 0.065, *P* = 0.0014; and tumor model NRC, 0.119 ± 0.053, *P* = 0.025, respectively; Figure 6C,E). In fact, when comparing between treatment arms in both tumor models, 24h post‐RT THb values normalized to pre‐RT scan were greater in SDRT‐treated groups compared to controls, although not significantly.

LME modeling revealed that ${\mathrm{sO}}_{2}$ changed modestly in our dataset, and only significantly from baseline between the different tumor models (tumor model NRC, 0.071 ± 0.036, *P* = 0.046; Figure 6D,F). No significant ${\mathrm{sO}}_{2}$ changes were measured 24h post‐RT. Nonetheless, sO_2_ quantified on the 24h post‐RT scans was indicative of treatment response across groups when conducting bivariate correlation analysis with endpoint IHC markers (Figure S4). sO_2_ at 24h post‐RT also showed strong correlation with change in tumor volume, proliferation, and DNA damage (change in tumor volume *r* = –0.47, *P* = 0.014, Ki67 *r* = –0.55, *P* = 0.0053, and γ‐H2AX *r* = 0.54, *P* = 0.0051). Moreover, mean sO_2_ negatively correlated with endpoint hypoxia (HIF1‐α *r* = –0.42, *P* = 0.0188), while its SD was also predictive of endpoint decrease in tumor size (*r* = 0.46, *P* = 0.034). SD of sO_2_ can be appreciated in the sO_2_ density distribution subplots of Figure 6A,B. Thus, PAI tomography could capture early increases in intratumoral blood content, which are likely related to radiation‐induced transient inflammatory activity following high doses of radiation, and longitudinal oxygenation mapping was found to be indicative of endpoint tumor control.

**FIGURE 7:**
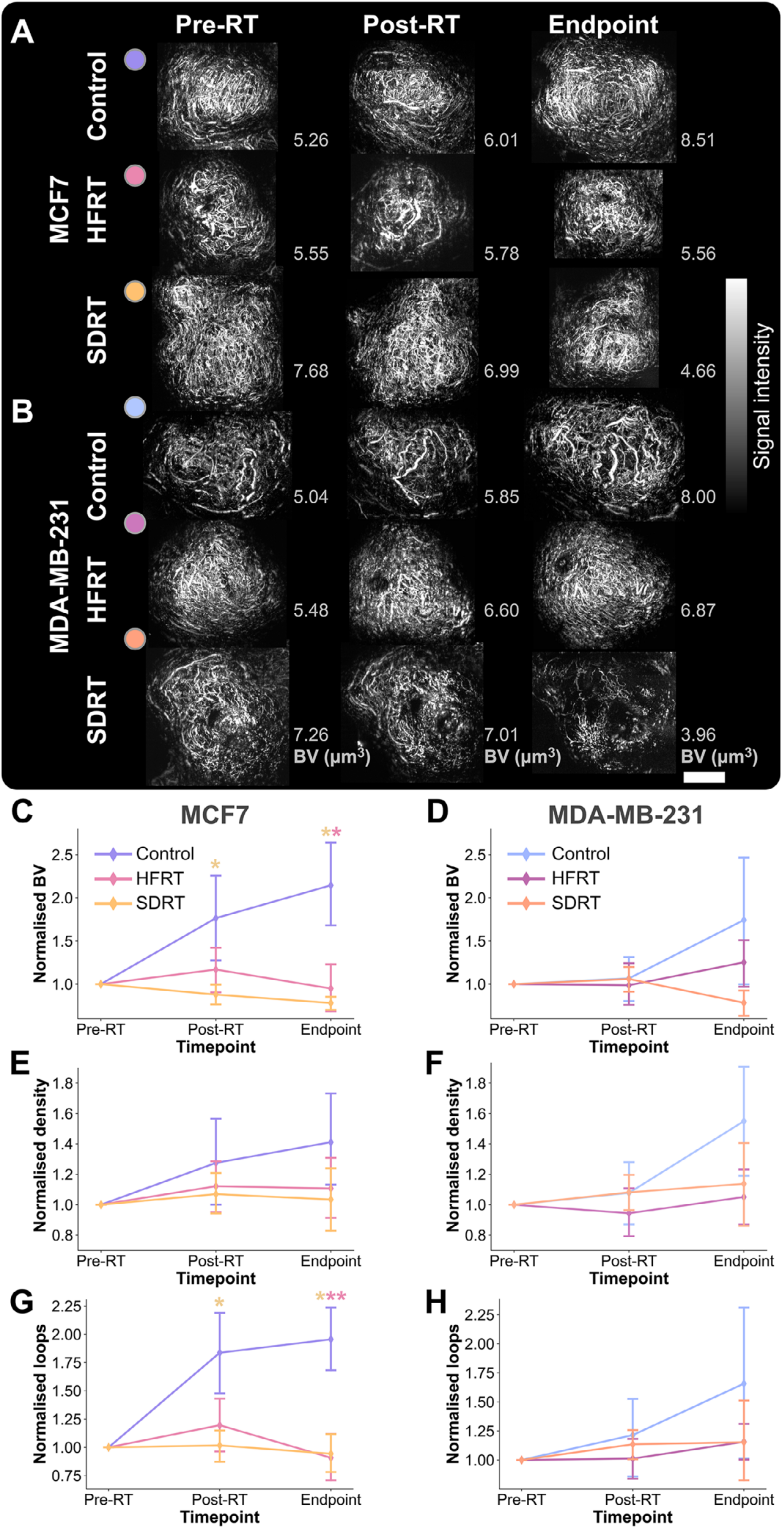
Longitudinal in vivo mesoscopic photoacoustic imaging of treatment response in breast cancer models. Exemplar maximal intensity projections of vascular networks on photoacoustic mesoscopy in (A) MCF7 and (B) MDA‐MB‐231 xenografts, across time‐points and treatment groups. Scale bar, 4 mm. Mesoscopic photoacoustic imaging biomarkers normalized to pre‐treatment scan in MCF7 and MDA‐MB‐231 tumor‐bearing mice, respectively, (C,D) blood volume (BV), (E,F) vascular density, and (G,H) looping structures. All imaging biomarkers in panels (C–H) are normalized to 1 at the pre‐RT time‐point. Point and bars represent mean and standard deviation in MCF7 groups (control *n* = 6, HFRT *n* = 9, or SDRT *n* = 8) and MDA‐MB‐231 groups (control *n* = 7, HFRT *n* = 6, or SDRT *n* = 10). Exact endpoint days are provided in Table S1. *P*‐values are calculated using two‐tailed Mann–Whitney *U*‐test with Holm–Bonferroni correction: *P < 0.05, **P < 0.01, ***P < 0.001.

As depicted on mesoscopic PAI exemplars (Figure 7A,B), blood volume decreased most in SDRT‐treated mice, then in HFRT‐treated groups, and was altered with increasing time‐points according to LME analysis (SDRT arm NRC, –0.500 ± 0.144, *P* = 0.001; HFRT arm NRC, –0.405 ± 0.150, *P* = 0.007; and time‐points NRC, 0.111 ± 0.055, *P* = 0.045). Similarly, vascular density and connected components changed most over time, as shown by LME modeling (time‐points NRC, 0.082 ± 0.034, *P* = 0.015; 0.188 ± 0.050, *P* = 0.0002; SDRT arm NRC, –0.373 ± 0.143, *P* = 0.009; and HFRT arm NRC, –0.326 ± 0.149, *P* = 0.029). Normalized looping structures were decreased in both treatment groups and changed with time‐points (HFRT arm NRC, –0.384 ± 0.152, *P* = 0.012; SDRT arm NRC, –0.373 ± 0.146, *P* = 0.011; and time‐point NRC, 0.134 ± 0.054, *P* = 0.014). Average diameters extracted from longitudinal mesoscopic PAI did not reveal any difference between models, treatments or time‐points.

Blood volume was decreased in the MCF7 xenograft model within 24h post‐RT in SDRT‐treated mice, before any change in tumor size was observed (normalized BV, *P* = 0.020; Figure 7C), but not in MDA‐MB‐231 (Figure 7D). At this early time‐point, the vascular density was also decreased in both models in treated groups, but not significantly (Figure 7E,F). In the more radiosensitive MCF7 model, the number of looping structures was significantly decreased compared to control in SDRT‐treated mice (*P* = 0.029; Figure 7G), thus decreasing the overall tortuosity of the vascular network, which was not observed in the more radioresistant MDA‐MB‐231 xenografts (Figure 7H). The observation of significant BV differences only in MCF7 tumors treated with ablative doses of radiation likely indicates endothelial cell damage at high doses. Finally, the measured BV 24h post‐RT was predictive of Ki67 positive areas at endpoint (*r* = 0.32, *P* = 0.0384; Figure S4), and BV and loops (normalized to BV) were both correlated with hypoxia (HIF1‐α *r* = –0.34, *P* = 0.0487, and *r* = –0.58, *P* = 0.00013; respectively). Thus, PAI mesoscopy provided early detection of response in the tumor vasculature, prior to any change in tumor size, indicating its sensitivity to radiation‐induced vessel pruning in the radiosensitive model.

### 2.4 Differential Vascular Response to Radiation Therapy Schemes Revealed by Photoacoustic Imaging at Endpoint

At endpoint, seven days on average following the last day of treatment, growth was significantly delayed in treated groups of both investigated breast cancer models (Figure 2). A final imaging session was conducted prior to tumor excision for ex vivo analysis. THb increased between pre‐RT and endpoint in SDRT‐treated MCF7 and MDA‐MB‐231 xenografts compared to their respective controls, and while the opposite trend was observed with HFRT‐treated groups (Figure 6C,D), neither reached statistical significance. ${\mathrm{sO}}_{2}$ was higher in treated MCF7 tumors compared to controls, but only significantly in HFRT‐treated mice (*P* =0.017), while no significant difference was observed in the more radioresistant MDA‐MB‐231 model. In terms of imaging‐IHC correlations, THb was negatively correlated with hypoxia (HIF1‐α *r* = –0.45, *P* = 0.038).

On mesoscopic PAI, the change in BV from baseline was significantly lower in MCF7 tumor‐bearing treated groups compared to controls (HFRT, *P* = 0.028; and SDRT, *P* = 0.017; Figure 7A), and was also lower in treated MDA‐MB‐231 xenografts but not significantly (HFRT, *P* = 0.35; and SDRT, *P* = 0.082; Figure 7B). The same trend was observed in measured blood vessel density in both MCF7 and MDA‐MB‐231 xenografts, but was not significant. The normalized count of looping structures was significantly reduced at endpoint in treated MCF7 tumors only (HFRT, *P* = 0.0079; SDRT, *P* = 0.014). Interestingly, the group averages of total BV change from baseline decreased in the same fashion as the CD31‐ASMA percent area in both models, with higher quantified values in control, HFRT and then SDRT (Figure 3A,B). BV was also correlated with the change in tumor size (*r* = 0.53, *P* = 0.0042; Figure S4). CC and density at endpoint were both positively correlated with proliferation assessed on Ki67 IHC (*r* = 0.48, *P* = 0.0052, and *r* = 0.42, *P* = 0.023; respectively) and CC were also negatively correlated with the extent of DNA damage (γ‐H2AX *r* = –0.42, *P* = 0.035). Taken together, our findings indicate a higher volume of denser functional blood vessels at endpoint is correlated with poorer overall tumor response, or radioresistance, in the investigated breast cancer models.

## 3 Discussion

**FIGURE 8:**
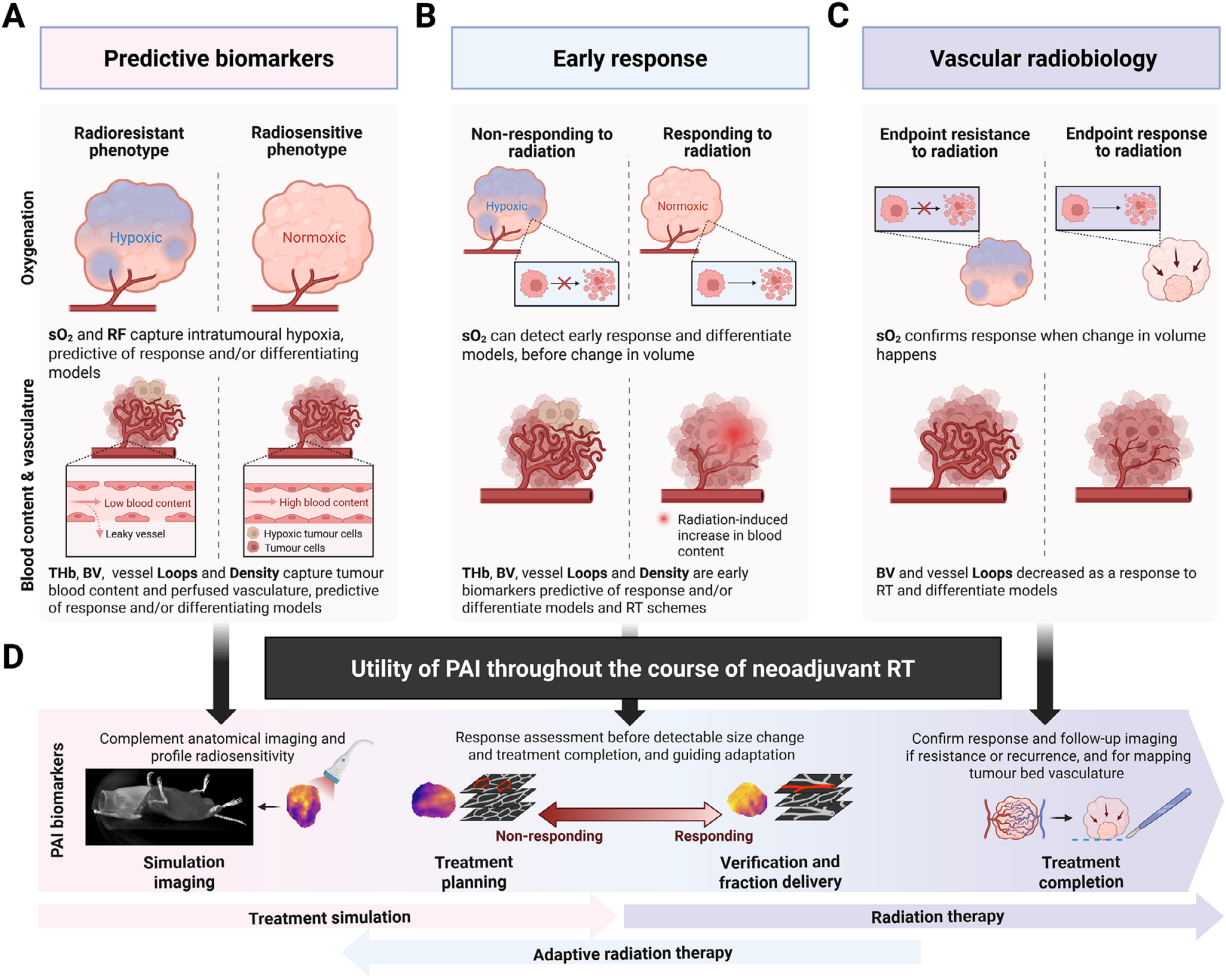
Conceptual schematic of reported PAI biomarkers for radiation response assessment in the tumor vascular microenvironment of breast cancer models. (A) PAI biomarkers 24h pre‐RT are predictive of response in terms of endpoint change in volume, DNA damage, and proliferation; (B) and remain predictive and identify early response 24h post‐RT. (C) Endpoint imaging reveals the vascular response to RT with endothelial cell apoptosis leading to microvascular disruption, identified as a decreased loop count and overall blood volume. (D) Potential use of PAI within the neoadjuvant RT framework, at baseline for profiling tumor radiosensitivity, for mapping intratumoral oxygenation to enable dose painting and further adaptation over the course of treatment, the ability to detect and predict response, and value in confirming response following treatment completion for improved tumor resectability guidance. Note: sO_2_, blood oxygen saturation; RF, responding fraction; THb, total haemoglobin; BV, blood volume; PAI, photoacoustic imaging; RT, radiation therapy. Created with BioRender.com.

The challenge of treating hypoxic solid tumors with RT has long been recognized, yet is rarely acted upon in treatment practice. With the increasing use of hypofractionation, hypoxia becomes a more critical consideration, and there is a clear need to develop suitable imaging biomarkers to detect and monitor changes in tumor vascular architecture and function in response to RT treatment. Biomedical optics has been identified previously for their versatility in capturing noninvasively endogenous molecular contrast for radiation oncology applications and at depth when combined with ultrasonic detection in PAI systems [38, 56, 57]. In this study, following the implementation of dosimetric quality assurance protocols [58], we sought to demonstrate the potential of PAI in this context, comparing hypofractionated and ablative single dose RT schemes in breast cancer xenografts exhibiting differential radiation responses showing wide‐ranging potential for PAI guidance of RT (Figure 8).

Tumor growth kinetics and ex vivo analysis of the two models aligned with the literature, showing that the slower growing MCF7 tumors have a more developed and mature vasculature, associated with lower hypoxia [55, 59] and higher radiosensitivity [47, 48, 49, 50, 60]. Both models showed gradual reductions in vessel maturity from control, to HFRT and then SDRT arms, alongside a characteristic decrease in hypoxia through HIF1‐α IHC in SDRT‐treated groups. Prior work in the same models showed increased CAIX on IHC in MDA‐MB‐231 tumors in comparison to MCF7 tumors [55]. Here, HIF1‐α differences trended similarly but were not significant, likely reflecting the marker biology and the transient nature of hypoxia, where HIF1‐α has a shorter half‐life (<10 min) while the downstream CAIX has a much larger half‐life (≈ 40h), thus influenced by the timing of our endpoint [61, 62, 63].

In vivo imaging biomarkers evaluated in this study revealed pre‐RT differences between the MCF7 and MDA‐MB‐231 models in terms of tumor blood content, intratumoral oxygen diffusion, and vascular morphology, aligned with prior work [55]. It should be noted that the biomarkers studied here reflect only the oxygenation status and concentration of haemoglobin, rather than being a direct assessment of tissue oxygenation or oxygen diffusion. We found that increased THb and decreased heterogeneity of oxygen saturation (SD of sO_2_) prior to treatment were associated with tumor control at endpoint across both models. It is worth noting that although MDA‐MB‐231 have been reported to have a higher microvascular density than MCF7 on IHC [55, 59], the mesoscopic PAI measurements demonstrated a significantly denser network in the MCF7 model, even though the total blood volume did not differ. This discrepancy could be explained by the fact that mesoscopic PAI depicts only the blood‐filled vessels at the peripheral rim of the tumor and can only resolve vessels from one another if they are larger than its in‐plane resolution (≈ 20 μ m), whereas IHC analyses the core and samples all vessels expressing endothelial cell proteins. Of note, a previous study has shown that orthotopic breast cancer tumors used in our study produce more vascularized tumors compared to subcutaneous tumors, used in prior studies [64]. Encouragingly though, increased blood volume pre‐RT in mesoscopic PAI correlated with endpoint response, similar to whole tumor‐evaluated THb on tomographic PAI. Nevertheless, both PAI configurations exploit endogenous haemoglobin contrast; however, they differ in spatial resolution and therefore in the information recovered. Tomographic PAI provides whole‐cross‐section measures of blood content, whereas mesoscopic PAI resolves individual superficial vessels at the tumor periphery, enabling the extraction of morphological vessel metrics.

Others have reported tumor blood oxygen saturation (sO_2_) pre‐treatment as a predictive biomarker for RT response in patient‐derived xenografts of head & neck cancer or pancreatic cancer models [44, 65, 66]. However, even though sO_2_ was higher on average in the more radiosensitive MCF7 at baseline, we did not observe a significant difference in our study; sO_2_ was only significantly different at endpoint. Most interestingly, we were able to assess in vivo the well‐known oxygen‐enhancement effect in RT [10], by finding pre‐RT a correlation between sO_2_ as measured with PAI tomography and the extent of DNA damage at endpoint, which was consistent across models and treatment groups at the individual level. Our results confirm those of a previous study using nanosonophores as a PAI contrast agent for oxygen sensing, which also showed that pre‐RT oxygen distribution was associated with post‐RT DNA damage ex vivo on γ‐H2AX in breast cancer patient‐derived xenografts [67].

The apparent discrepancy between the predictive value of mean sO_2_ and SD of sO_2_ in our study likely reflects the fact that absolute mean oxygenation captures only a global estimate of vascular supply, whereas spatial heterogeneity in oxygenation provides a more integrative measure of functional perfusion uniformity and diffusion efficiency across the tumor mass. Tumors exhibiting high average sO_2_ but marked heterogeneity may still contain chronically or acutely hypoxic subregions that drive radioresistance, leading to poorer overall control despite higher mean oxygen levels. Conversely, a low SD of sO_2_ at baseline indicates more homogeneous perfusion and oxygen delivery, enabling more consistent radiosensitization throughout the tumor. In addition, γ‐H2AX reflects acute DNA damage following irradiation, whereas long‐term control depends on the capacity of clonogenic subpopulations in hypoxic niches to survive and repopulate. Therefore, the significant correlation between baseline vascular sO_2_ and γ‐H2AX likely captures the oxygen‐enhancement effect, while the stronger relationship between SD of sO_2_ and outcome reflects the lasting impact of oxygenation heterogeneity on treatment resistance and regrowth dynamics [1]. We also note that PAI‐derived sO_2_ is a surrogate biomarker of detectable oxygen levels within blood vessels mainly carried by heme group in red blood cells, and is not representative of dissolved extra‐ or intracellular oxygenation in tumor tissue.

Change in blood oxygen saturation (ΔsO_2_) and the responding fraction under gas challenge pre‐RT were were both significantly higher at baseline in the MCF7 groups than in the MDA‐MB‐231, indicating better intratumoral oxygen diffusion, linked to higher radiosensitivity in MCF7. A prior study investigating the same models revealed that blood oxygen level dependent‐MRI ΔR2*, a surrogate marker for oxygen delivery under gas challenge, showed improved blood oxygen diffusion in MCF7 compared to MDA‐MB‐231 xenografts, in‐line with our findings [59]. Increased tumor ΔsO 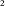 pre‐treatment was also demonstrated to be a predictive biomarker in pancreatic cancer models [66, 68], highlighting the potential of using a breathing gas challenge to extract predictive PAI biomarkers.

Importantly, PAI revealed regimen‐specific effects of ablative (SDRT) vs. hypofractionated (HFRT) RT in the two models. Since RT can transiently stimulate tumoral neovascularization and perfusion via acute inflammation [69] and can also prune hypoperfused vessels [70, 71, 72, 73], blood flow may increase shortly after irradiation, thereby increasing THb. Consistent with this, THb increased at 24h in SDRT‐treated tumors, indicating a transient vascular contribution to response. In fact, in the radiosensitive MCF7 model, mesoscopic PAI analysis showed reduced loop count 24h post‐RT and/or at endpoint in SDRT‐ and HFRT‐treated tumors, consistent with radiation‐induced pruning, which in turn improved intratumoral microvascular blood content captured through THb. Pruning of small dysfunctional vessels has been observed early in response to high dose RT in other cancer models [71, 72, 73]. A recent study explained through computational modeling that blood content may be increased following RT as a result of the pruning of hypoperfused vessels and loops [70], indicating that larger blood vessels and increased network complexity does not provide improved efficient blood flow. Taken together, the combination of tomographic and mesoscopic PAI in our dataset showed that a high residual denser superficial blood volume was associated with poorer response.

Oxygenation changes were modest and model‐ and regimen‐dependent: in MCF7, endpoint sO_2_ was higher only in the HFRT arm vs. control, with no difference between SDRT and control; in MDA‐MB‐231, no between‐group differences in longitudinal sO_2_ measurements were detected. Nevertheless, higher sO_2_ at 24h post‐RT correlated with improved endpoint control across arms. Taken together, the sustained sO_2_ increase observed exclusively in HFRT‐treated radiosensitive tumors (MCF7) is most plausibly explained by reduced oxygen consumption from damaged tumor cells, as corroborated by higher γ‐H2AX and lower Ki67, rather than increased supply or diffusion, whereas the absence of an sO_2_ rise after SDRT, despite a transient THb increase, suggests that early microvascular flow impairment does not impact oxygen delivery in this setting. This pattern is consistent with the radiobiological principle that fractionation enables reoxygenation between fractions, through partial vascular recovery and reperfusion, reduced interstitial pressure, and lowered oxygen consumption in damaged cancer cells, thereby increasing intratumoral vascular sO_2_ [13]. Increased sO_2_ post‐RT has also been observed by others, but may also likely be impacted by differences in model, dose/timing of RT delivery, or imaging configuration [46, 74, 75]. By the classic four R's of radiobiology, fractionation also enables redistribution of surviving tumor cells between fractions, favoring a more durable suppression of proliferation than a single ablative fraction, which may underpin the non‐significant difference in Ki67 on IHC in SDRT‐treated MDA‐MB‐231 tumors [13].

Despite these promising findings, there are limitations to our study. A first key limitation is that the imaging modalities investigated here only provide surrogate biomarkers of complex biological processes focusing on tissue vascularization, blood content and oxygenation, and not cell death or immune response, and must therefore be carefully interpreted. For instance, tomographic PAI‐derived blood oxygen saturation is not an absolute oxygenation measurement, but provides insightful relative sampling of the intratumoral oxy‐ and deoxyhaemoglobin with high technical reproducibility (< 4% variability) [76, 77]. Similarly, mesoscopic PAI enables only the characterization of superficial (≈ 4 mm) perfused vasculature in vivo. The full extent of the investigated tumor vascular networks – especially in perfusion‐limited vessels – is not fully captured in this detection configuration. For instance, due to the top illumination and planar detection geometry, vessels approaching 90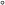 to the detection plane are not detected, creating discontinuities in the network and artificially increasing the number of connected components [78]. Thus, the quantitative biomarkers presented in this study cannot be taken outside their context or as absolute measurements, and do not fully recapitulate the complex dynamic response to RT. While treated tumors showed significant growth delay at endpoint, our post‐RT sampling was not designed to robustly assess the impact of differential growth rates in selected models, and rather focused on probing the vascular microenvironments in size‐matched tumors to establish those imaging biomarkers. Beyond tumor volume, vascular, and oxygenation changes, post‐RT immune mechanisms are increasingly recognized as critical modulators of tumor control [63, 79]. Integrating markers of immune activation, such as cytotoxic T‐cells and macrophages in future studies with extended follow‐up could help elucidate the interplay between growth kinetics, vascular remodelling, hypoxia and anti‐tumor immunity, combining PAI with multiplex immunofluorescence protocols for instance. Tailored contrast agents that have been introduced recently could be employed for identifying specifically cellular response mechanisms such as senescence [80, 81].

A second key limitation is that we have not completed serial culling of animals to obtain tumor samples at all imaging time‐points of interest. Instead, we favored having paired longitudinal imaging data to improve statistical power, thus decreasing the need for high animal numbers per group. Such ex vivo IHC analysis would have provided time‐resolved validation of our in vivo imaging. Furthermore, we did not keep animals alive longer than 7 days post‐RT on average to further monitor tumor growth, vascular alterations, or survival. Thus, considering the time‐scale of tumor and vascular response to RT, our imaging‐based investigation depicts the earlier phase of response that may not recapitulate the full story, especially in terms of recurrence or revascularization post‐RT [82]. Nevertheless, targeted neoadjuvant RT did improve resectability at endpoint, given the decrease in tumor size.

Finally, the imaging time‐points in terms of days between treated groups were different due to the nature of the hypofractionated (HFRT) vs. single‐dose (SDRT) courses of treatment. Even though treatment arms are compared directly, they are only comparable in terms of total dose delivered but not in absolute number of days. These differences could introduce a source of error due to the transient nature of hypoxia. Further study would be required to control for the time effect more directly. We believe that changes in intratumoral blood oxygen saturation following RT have an important dose‐ and time‐dependence, which could be tumor‐specific, as highlighted in our comparisons with prior literature. We acknowledge that there is a time compartment to radiation response, but report here differences related to the impact of fractionation and dose per fraction.

In terms of clinical translatability, although neoadjuvant RT is not the main line of treatment in breast cancer patients, it has been shown to be beneficial in low‐risk or early‐stage breast cancer, including for ER+ cases with potential chemo‐resistance [83, 84, 85]. Indeed, there has been an increasing interest in breast cancer pre‐operative hypofractionated RT or even SDRT, enabled by the increased precision in tumor targeting provided by recent developments in integrated imaging and accelerator technologies (e.g. magnetic resonance‐guided RT, PET‐guided – also termed biology‐guided – RT, or camera‐guided CyberKnife) that decrease overall treatment time [86]. Pre‐operative partial breast irradiation using 20 Gy SDRT to the tumor volume, similar to that described in our preclinical study, has been investigated and shown oncologically safe in a feasibility study and a 5‐year follow‐up study [87, 88], and in another trial using 21 Gy SDRT [89], and are both being further investigated in on‐going clinical trials (ClinicalTrials.gov ID, NCT03863301, NCT02212860, and NCT04679454; Australian New Zealand Clinical Trials Registry, ACTRN12621000659808) [90, 91]. The potential of PAI in this context is multi‐faceted, and clinical studies should investigate the use of quantitative PAI in breast cancer patients receiving RT or combination therapies [38]. For instance, preclinical data in numerous cancer models already support the benefits of using functional imaging modalities to guide the boosting of the radiation dose to tumor hypoxic fraction, using oxygen‐guided spatially modulated dose delivery [14, 92]. Moreover, a recent clinical study in head & neck cancer patients demonstrated the feasibility of longitudinal bedside PAI to track RT‐related changes in tumor‐adjacent tissues such as lymph nodes, supporting the clinical translation of functional PAI for treatment monitoring [93]. Thus, the role of label‐free portable bedside clinical photoacoustic imagers in the radiation oncology clinics should be further investigated, especially in the context of treatment adaptation.

## 4 Conclusion

We demonstrated that PAI reveals tumor radiosensitivity and the differential effect between hypofractionated and ablative courses of RT in two breast cancer models, with increased blood content and gas challenge response pre‐RT associated with improved treatment outcomes. We showed that PAI could capture early RT response, days before any change in tumor volume was measured, informing on radioresistance. The vascular alterations resulting from ablative doses of radiation were seen within 24h of treatment in the more radiosensitive model. By capturing early oxygenation gain under hypofractionation and microvascular disruption after ablative dosing, multi‐scale PAI provides readouts of RT response that can precede tumor volume regression, supporting its translational promise for early response‐guided adaptation. Hence, the molecular contrast provided by PAI shows promise for measuring functional response and shedding new light on the vascular compartment of radiation response, as well as the impact of hypoxia on radioresistance in vivo. We envision that PAI biomarkers will benefit other cancer types treated with RT, such as already demonstrated preclinically in head & neck cancer [44, 45], with high translational potential in those more superficial tumor sites. Visualizing and quantifying the tumor vascular phenotype in vivo thus has the potential to enable personalized RT through biology‐tailored targeting and further inform the course of treatment.

## 5 Experimental Section

### 5.1 In Vitro Cell Line Preparation

Two human breast adenocarcinoma cell lines were selected as part of this study: MCF7 (Research Resource Identifier [RRID]:CVCL_0031), a luminal A subtype with estrogen and progesterone receptors positive, and human epidermal growth factor receptor 2 (HER2) negative (ER+, PR+, HER2‐), and MDA‐MB‐231 (RRID:CVCL_0062), a triple negative (ER‐, PR‐, HER2‐) mesenchymal stem‐like basal B subtype invasive ductal carcinoma (Figure 1A). The models exhibit differential response to RT in vitro (survival fraction at 2 Gy of 0.70–0.82 in MDA‐MB‐231 cell cultures and 0.40–57 in MCF7 cell cultures [47, 48, 49, 50]) and when implanted as xenograft models give distinct intratumoral hypoxic and vascular features for in vivo PAI [55] (Figure 1B,C). Human cell lines were obtained from the local biorepository of the Cancer Research UK Cambridge Institute at the University of Cambridge, UK, originally acquired from the American Type Culture Collection (ATCC, HTB‐22 and HTB‐26). Cells were used only between passages 25–27 (MCF7) or 35‐37 (MDA‐MB‐231). At the start of the study, authentication using Genemapper ID (v3.2.1, Genetica) by short tandem repeat (STR) profiling (1/2015) showed 100% match with reference sequences in the two cell lines. All cell lines tested negative for mycoplasma contamination prior to use and were regularly monitored throughout the study. Cell lines were passaged and maintained separately in Roswell Park Memorial Institute (RPMI) 1640 Medium (RPMI 1640, Gibco, ThermoFisher Scientific) supplemented with 10% of Foetal Bovine Serum (FBS, Gibco, ThermoFisher Scientific) at 37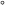 C in 5% ${\mathrm{CO}}_{2}$, never exceeding 80% confluence.

### 5.2 Animal Models

Scientific procedures on small animals were performed under the authority of project (PE12C2B96) and personal licenses issued by the Home Office, UK, under the Animals (Scientific Procedures) Act, 1986, and were reviewed for compliance by the local Animal Welfare and Ethical Review Board (compliance form CFSB2226) at the Cancer Research UK Cambridge Institute. All procedures were conducted following the latest guidance on animal welfare [94]. Well‐being was monitored daily by animal technicians and study conductors. Animals were housed in sterile conditions within hermetic individually ventilated cages with efficiency particulate air filtration, on 12h on/12h off light cycles, and were provided with free access to irradiated sterile food (Mouse Diet 20 Extruded 5R58, PicoLab) and water.

After habituation, subcutaneous cell implantations were performed in the location of the mammary fat pad in eight‐week‐old immunodeficient female BALB/C nude mice (BALB/c nu/nu, Charles River) (Figure 1D). Cell lines were tested for absence of mycoplasma contamination prior to animal implantation. Inoculations of 1 × 10^6^ cells were prepared in a 50 μ L solution with a 1:1 ratio of basement membrane extract (Cultrex BME, PathClear, R&D Systems, Bio‐Techne) and phosphate‐buffered saline (PBS, Gibco, ThermoFisher Scientific), kept on ice. For the ER+ cell line (MCF7) tumor‐bearing mice, estrogen pellet implantations (E2‐M 17β‐estradiol 90‐day implants, Belma Technologies) were conducted 48h prior to inoculations by surgically implanting a single pellet in the scruff of the neck of animals according to recent protocols [95]. When tumors reached a measurable size (> 3 mm in diameter), calliper measurements and animal weighing were performed up to 3 times per week for monitoring tumor growth and animal health. Diameter was measured in the two major orthogonal axes of the growing mass, a, the smallest axis, and b, the largest axis, and tumor volume, V, was calculated as $V=ab^{2}\pi /6$. Percent changes in volume between enrolment and endpoint were calculated as $\Delta V\left(\%\right)=\left(V_{end}-V_{enr}\right)/\left(V_{end}+V_{enr}\right)$. Humane endpoints were defined as: i) tumor reaching over 1.5 cm in average diameter; ii) a weight loss surpassing 15% of enrolment weight; iii) acute clinical signs of ill health such as lack of movement, distress, and poor body score; iv) skin conditions, such as moist desquamation or bleeding ulceration; and v) in hormone supplemented animals, persistent skin lesions or bladder calcification. During treatment, if signs of weight loss were observed, dietary supplementation in the form of recovery gel (ClearH_2_O DietGel Recovery, Datesand Group) could be provided to animals for their demonstrated value in supporting recovery [96]. If hormone supplementation related adverse effects were observed in MCF7‐tumor bearing xenografts, animals were closely monitored up to twice a day, applying skin cream (DermaGel) and/or providing bladder massages to facilitate urine passage and limit the potential development of calcification, in consultation with the local named veterinary surgeon.

### 5.3 Radiation Therapy Planning and Delivery

All preclinical image‐guided RT experiments were conducted using the small animal radiation research platform (SARRP, Xstrahl) mirroring clinical RT standards [51]. RT was delivered under X‐ray cone‐beam computed tomography (CBCT) guidance in the local TG‐61‐calibrated SARRP with moving arcs optimized to target tumors and spare healthy tissue [97]. A summary of differences between preclinical and clinical RT is provided in Table S2.

Mice were enrolled onto a treatment course of one of the two selected biologically‐equivalent RT schemes, when tumors reached a volume of 400 mm^3^: single dose RT (SDRT) of 20 Gy, tested recently in multiple clinical trials in breast cancer patients [87, 88, 89, 90, 91], or hypofractionated RT (HFRT) of 25 Gy in 5 fractions of 5 Gy, mirroring the dose fractionation of recent clinical trial in breast cancer patients [98] but in a neoadjuvant context (Figure 1D–F). A radiobiological comparison of selected RT schemes is provided in the Supporting Information. Before enrolment into one of the treatment or control groups, all mice were imaged using PAI 24h before the first day of treatment (Day 0, Figure 1F). Then, tumor‐bearing mice from the MCF7 model (*n* = 23) and MDA‐MB‐231 (*n* = 23) were blindly randomized into control or treated groups (Control, *n* = 13; SDRT, *n* = 18; HFRT, *n* = 15), receiving treatment over 1 day for SDRT or 5 days for HFRT. Post‐RT imaging was conducted 24h after the last RT fraction in each scheme (Day 2 for SDRT, Day 6 for HFRT; Figure 1F) and at endpoint. Mice reached endpoint 7 days post‐RT on average (Day 8 for SDRT, Day 12 for HFRT). Summaries of the treatment and imaging schedules for all animals are provided in Table S1.

### 5.4 In Vivo Photoacoustic Imaging

In vivo tumor imaging was conducted in anaesthetized mice using mesoscopic (RSOM; Explorer P50, iThera Medical GmbH) and tomographic (MSOT; inVision, iThera Medical GmbH) PAI systems 24h pre‐RT, 24h post‐RT and at endpoint for all mice. Prior to imaging, anaesthesia was induced in animals using 3% isoflurane in a gaseous mix of 50% medical air and 50% pure oxygen. Mice were then transferred to the mesoscopic PAI system on a heated bed kept at 37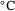 in supine position. Breathing was monitored and maintained between 70–80 breaths per minute, decreasing anaesthetic concentration to 1.5‐2% isoflurane after induction, and maintained throughout imaging. The transducer head was manually positioned on the tumor region and coupled to the skin surface using warmed centrifuged ultrasound gel (Aquasonic Clear, Parker Laboratories Inc.). After careful removal of any residual air bubbles, raster‐scanning PAI acquisitions were conducted to capture perfused peripheral tumor vasculature.

Anaesthetized animals were then moved to the tomographic PAI system animal holder and a membrane coupled to their skin with warmed ultrasound gel. Multispectral acquisitions were performed to reveal functional biomarkers of total blood content and oxygenation in vivo. A breathing‐gas challenge was performed during tomographic PAI: time‐resolved scans were acquired during 5 min on medical air (21% O_2_), after which the breathing gas was switched to pure oxygen (100% O_2_) for another 5 min as described previously [42] (exemplar quantified images in breathing gas challenge in Figure S5).

For pre‐RT and post‐RT scans, mice were slowly recovered in a heated chamber until active and inquisitive. After the endpoint scan, mice were euthanized with a recognized Schedule 1 method and death was confirmed prior to surgical tumor resection for histopathological processing.

### 5.5 Photoacoustic Image Analysis and Quantitative Imaging Biomarkers Extraction

All image analyses were conducted in Python 3.9. Mesoscopic PAI captured the tumor vasculature in a 12 × 12 mm^2^ field of view and up to a depth of 4 mm, thus provided a detailed view of the tumor periphery. Mesoscopic photoacoustic images were segmented using the vessel segmentation generative adversarial network (VAN‐GAN) [54]. Segmented vascular networks were skeletonized, and the total blood volume (BV, μm ^3^), the number of perfused vascular connected components (CC, representing the number of sets of vertices connected by path in the vascular skeleton), the vessel segments density (μm ^−3^), the average diameters (μm), and the count of looping structures normalized to blood volume (loops, representing a collection of vertices connected by edges in the vascular skeleton forming a path closed on itself, μm ^−3^) were extracted as quantitative imaging biomarkers, using previously reported software packages [73, 99] (mesoscopic PAI metrics summary provided in Table S3).

Multispectral tomographic PAI was used to capture functional biomarkers using the Python photoacoustic tomography analysis toolkit (PATATO) [53] Oxy‐ and deoxy‐haemoglobin signals were quantified to extract mean total haemoglobin (THb), blood oxygen saturation (sO_2_), and the standard deviation (SD) of sO_2_. Gas challenge tomographic data was analysed using sO_2_ time‐series data to quantify the change in sO_2_ (ΔsO_2_) under gas challenge in the tumor, and the responding fraction (RF). Further details are provided in the Supporting Information.

### 5.6 Ex Vivo Histopathological Analysis

Excised tumors were processed, and formalin‐fixed paraffin‐embedded (FFPE) sections were taken for immunohistochemical (IHC) staining and analysis. Briefly, after excision, tumors were fixed in 10% formalin for 24h, and then moved to 70% ethanol for 48h. Resected tumors were then embedded in paraffin, sectioned, and rehydrated. IHC was performed using an automated stainer (BOND, Leica Biosystems) with a bond polymer refine detection kit and 3,3'‐diaminobenzadine as a substrate. Tissue sections were stained for endothelial cell marker CD31, smooth muscle cell marker α‐smooth muscle actin (ASMA), cellular proliferation nuclear marker Ki67, phosphorylated histone nuclear marker of DNA damage γ‐H2AX, and hypoxia‐inducible pro‐angiogenic factor marker HIF1‐α (antibody vendor, dilution and retrieval method provided in Table S4). Adjacent 3 μm serial sections were used for CD31 and ASMA staining. Haematoxylin and Eosin (H&E) staining was performed using an automated system (ST5020 Leica, Biosystems). Stained FFPE sections were scanned at 20× magnification using an Aperio AT2 with a resolution of 0.5 × 0.5 μm ^2^ (Leica Biosystems). Image analysis was conducted in HALO (v3.2, Indica Labs).

### 5.7 Statistical Analysis

All statistical analyses were conducted using the open‐source Pingouin (v0.5.5) and statsmodels (v0.14.3) packages in Python 3.9. Descriptive statistics of the distribution of PAI and IHC biomarkers were computed (mean ± standard deviation) and visualized with point and bar plots using the seaborn Python package (seaborn, v0.13.2). Unpaired non‐parametric two‐tailed Mann‐Whitney *U*‐test was used for comparing in vivo and ex vivo imaging biomarkers between groups and conditions with Holm–Bonferonni multiplicity correction (*m* = 5 comparisons, within tumor model, between control and treated arms, and between controls across models) [100]. For pre‐RT tumor model comparison, sample sizes were *n* = 23 for MCF7 and *n* = 23 for MDA‐MB‐231 tumor groups. For all other comparisons, sample sizes were *n* = 6 MCF7 control, *n* = 9 MCF7 HFRT, *n* = 8 MCF7 SDRT, *n* = 7 MDA‐MB‐231 control, *n* = 6 MDA‐MB‐231 HFRT, and *n* = 10 MDA‐MB‐231 SDRT (time‐point‐specific exclusions are shown in Table S1). Bivariate correlations between continuous variables extracted from in vivo PAI and ex vivo classified IHC regions were assessed with Pearson correlation coefficients (*r*), with associated Holm–Bonferonni‐adjusted *P*‐values. Quantitative imaging biomarkers measured at the different analysed time‐points of interest across mice and conditions were analysed as continuous variables with linear mixed effects (LME) models, using as fixed effects the following categorical variables: tumor model (MCF7 or MD‐MB‐231), treatment scheme (Control, HFRT, or SDRT), and ordinal time‐point (pre‐RT, post‐RT, or endpoint). LME‐estimated normalized regression coefficients, standard errors, and Holm–Bonferonni‐adjusted *P*‐values were reported for each biomarker. Significance level was set at α=0.05 for all adjusted *P*‐values.

## Author Contributions

TLL: conceptualization, data curation, formal analysis, funding acquisition, investigation, methodology, software, validation, visualization, writing – original draft. M‐EO: investigation, methodology, project administration, supervision, validation, writing – review & editing. EVB: investigation, methodology, validation, writing – review & editing. TRE: methodology, software, validation, writing – review & editing. LCW: investigation, validation, writing – review & editing. MAG: investigation, writing – review & editing. LH: investigation, validation, writing – review & editing. CB: formal analysis, investigation, methodology, software, writing – review & editing. SK: investigation, methodology, writing – review & editing. YC: investigation, methodology, writing – review & editing. LY: investigation, methodology, resources, project administration, writing – review & editing. PWS: formal analysis, investigation, methodology, software, supervision, validation, visualization, writing – review & editing. SEB: conceptualization, funding acquisition, methodology, project administration, resources, supervision, validation, writing – review & editing.

## Conflicts of Interest

The authors declare no conflicts of interest.

## Supporting information


**Supporting File**:advs74636‐sup‐0001‐SuppMat.pdf.

## Data Availability

All code and data associated with the results presented in this manuscript will be made available upon acceptance of the article at: https://doi.org/10.17863/CAM.118247.
