# Supplementary material for: Early Radiation Therapy Response Assessment Using Multi‐Scale Photoacoustic Imaging
Source: Adv Sci (Weinh). 2026 Mar 9;13(26):e09268. doi: 10.1002/advs.202509268 (PMC13159165; doi:10.1002/advs.202509268)
Supplement: Supplementary file 1 — Supporting File:advs74636‐sup‐0001‐SuppMat.pdf. [file ADVS-13-e09268-s001.pdf]

Supplementary Materials for: Early Radiation Therapy Response Assessment using Multi-scale Photoacoustic Imaging

Thierry L. Lefebvre[1,2], Mariam-Eleni Oraiopoulou[1,2], Ellie V. Bunce[1,2], Thomas R. Else[1,2], Lorna C. Wright[1,2], Monika A. Golinska[1,2,3], Lina Hacker[4], Cara Brodie[2], Steven Kupczak[2], Yi Cheng[2], Lisa Young[2], Paul W. Sweeney[1,2], and Sarah E. Bohndiek[1,2]✉

[1]Department of Physics, University of Cambridge, JJ Thomson Avenue, Cambridge, CB3 0HE, UK
[2]Cancer Research UK Cambridge Institute, University of Cambridge, Robinson Way, Cambridge, CB2 0RE, UK
[3]Department of Biostatistics and Translational Medicine, Medical University of Lodz, Al. Kosciuszki, Lodz, 90-419, Poland
[4]Department of Oncology, University of Oxford, Roosevelt Drive, Oxford, OX3 7DQ, UK

Supplementary Note 1: Supplementary Results

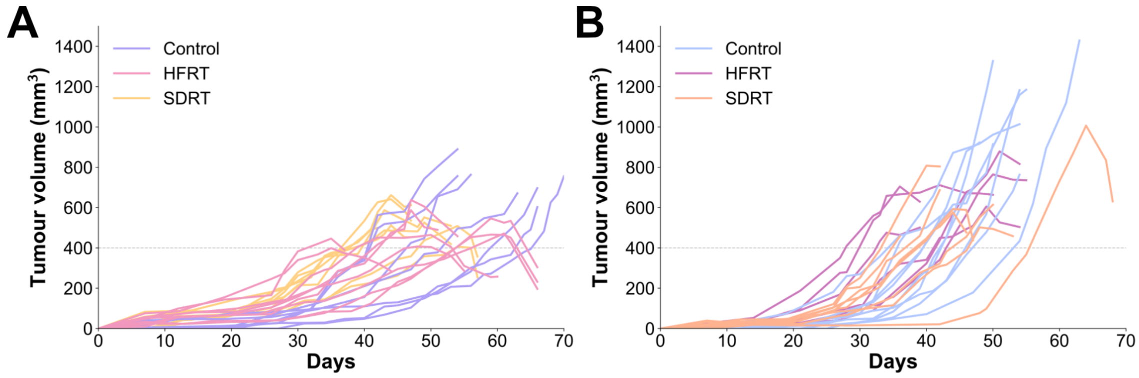

Supplementary Figure 1. Tumour growth curves in individual mice enrolled in the preclinical radiation therapy trial. Tumour volumes capture by calliper measurements from inoculation date to endpoint in A) MCF7 and B) MDA-MB-231 tumour-bearing xenograft models.

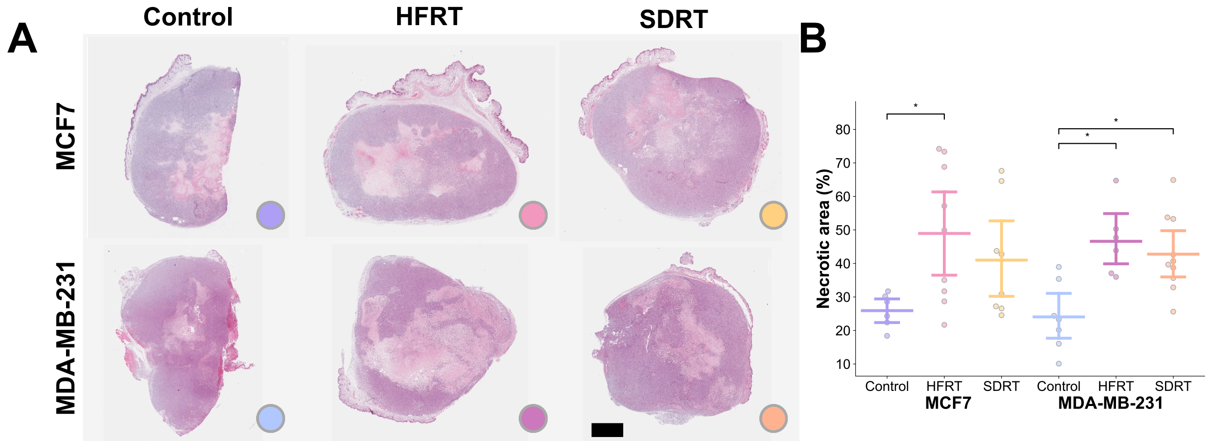

Supplementary Figure 2. Exemplar haematoxilin and eosin stain scans of resected tumour tissue sections in A) MCF7 (top panels) and MDA-MB-231 (bottom panels) breast cancer xenografts across treatment conditions. B) Quantified percent necrosis area across models and treatment groups in bar and point plots with bars representing mean and 95% confidence intervals. Scale bar, 1.5 mm.

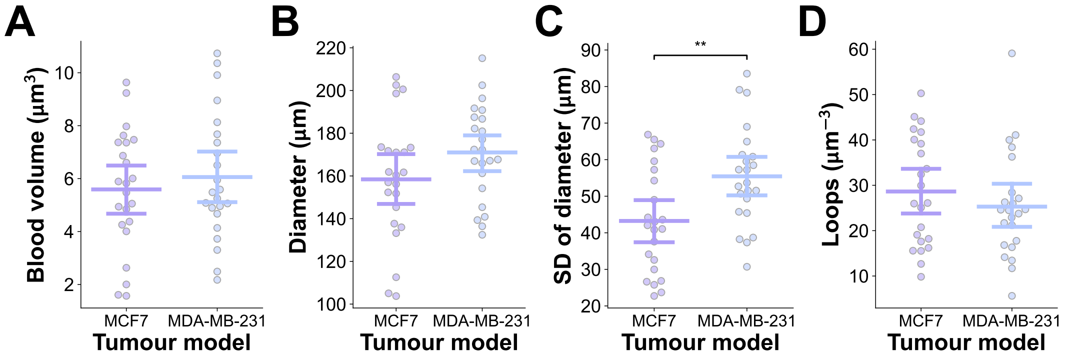

Supplementary Figure 3. Baseline mesoscopic photoacoustic imaging parameters quantified in segmented vascular networks. MCF7 and MDA-MB-231 xenografts' vasculature quantified for A) blood volume, B) average diameter, C) standard deviation of diameter, and D) loops normalised to blood volume in bar and point plots, with bars representing mean and 95% confidence intervals.

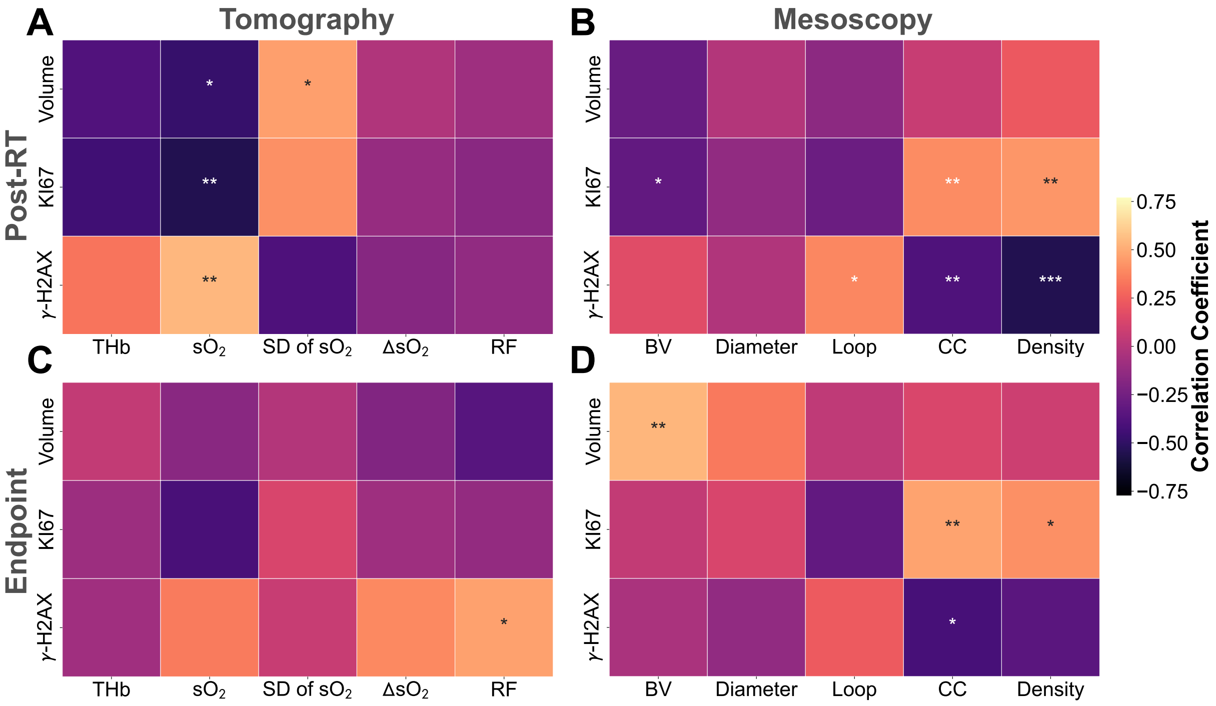

Supplementary Figure 4. Correlation of quantitative photoacoustic imaging biomarkers with endpoint immunohistochemistry. Correlation heatmaps of post-RT A) tomographic and B) mesoscopic PAI biomarkers, and of endpoint C) tomographic and D) mesoscopic PAI biomarkers with *ex vivo* immunohistochemistry parameters.

Supplementary Table 1. Detailed summary of animals included in the longitudinal preclinical radiation therapy trial. Enrolled mice engrafted with either investigated breast cancer cell lines and their imaging data inclusion or exclusion across acquisition time-points are reported. Colour-coding represent data acquired and included (green), acquired and excluded (yellow) and not acquired (red). Stars represent gas challenge data acquisition during tomographic photoacoustic imaging examination. Days post-radiation therapy after which mice were culled are reported. For the control group, days are counted from day 3 following enrolment on their post-radiation therapy imaging time-point. A total of 11 mice out of 46 had at least one reported exclusion. Note: BC, body condition; DoD, day of death; Excl., excluded; GC, gas challenge; ID, identifier; IHC, immunohistochemistry; No., number; PAI, photoacoustic imaging; RT, radiation therapy; Tx, treatment.

Tumour type	Tx arm	No.	Study ID	IHC	Mesoscopic PAI			Tomographic PAI			Exclusion reason	DoD post-RT
					Pre-RT	Post-RT	Endpoint	Pre-RT	Post-RT	Endpoint		
**MCF7**	SDRT	1	PAIinRT015					*	*	*		14
		2	PAIinRT016					*	*	*		14
		3	PAIinRT017					*	*		Weight loss	6
		4	PAIinRT032							*		7
		5	PAIinRT033							*		7
		6	PAIinRT034							*		7
		7	PAIinRT047					*	*	*		7
		8	PAIinRT048					*	*	*		7
	HFRT	9	PAIinRT019					*	*	*		14
		10	PAIinRT020					*	*	*		9
		11	PAIinRT025					*	*	*		7
		12	PAIinRT026					*	Excl.		Anaesthesia, GC	1
		13	PAIinRT035							*		7
		14	PAIinRT036							*		7
		15	PAIinRT049					*	*	*		7
		16	PAIinRT050					*	*	*		7
		17	PAIinRT051					*	*	*		7
	Control	18	PAIinRT039							*		7
		19	PAIinRT040							*		7
		20	PAIinRT052					*	*	*		7
		21	PAIinRT053					*	*	*		7
		22	PAIinRT054					*	*	*		7
		23	PAIinRT055					*	*	*		7
**MDA-MB-231**	SDRT	1	PAIinRT009					*	*		Inactive, poor BC	6
		2	PAIinRT010					*	*	*		14
		3	PAIinRT018					*	*		Weight loss	5
		4	PAIinRT022					*	*	*		8
		5	PAIinRT023					*	*		Inactive, poor BC	6
		6	PAIinRT024					*	*		Weight loss	6
		7	PAIinRT027					*	*		Anaesthesia	7
		8	PAIinRT028					*	*	*		7
		9	PAIinRT030							*	Poor BC	4
		10	PAIinRT031							*	Poor BC	4
	HFRT	11	PAIinRT012					*	*	*		14
		12	PAIinRT013					*	*		Weight loss	2
		13	PAIinRT014					*	*		Weight loss	2
		14	PAIinRT021					*	*	*	Ulceration	9
		15	PAIinRT037							*		7
		16	PAIinRT038							*		7
	Control	17	PAIinRT029					*	*	*		7
		18	PAIinRT041							*		7
		19	PAIinRT042					*	*	*		7
		20	PAIinRT043					*	*	*		7
		21	PAIinRT044					*	*	*		7
		22	PAIinRT045					*	*		Anaesthesia	7
		23	PAIinRT046		Excl.	Excl.	Excl.	*	*	*	Ulceration	7

Supplementary Note 2: Detailed Experimental Section

A. Radiation therapy using single arc delivery under imaging guidance. The differences to consider between preclinical and clinical radiation therapy (RT) are summarised in Supplementary Table 2. Using the small animal radiation research platform (SARRP, Xstrahl), cone-beam computed tomographic (CBCT) images were reconstructed for imaging guidance within a commercial treatment planning software (Muriplan, Xstrahl) from acquisitions of 360 projections at one-degree increments in imaging mode (peak energy, 60 kVp; beam filtration, 2.0 mm Al; maximum tube current, 2.8 mA; focal spot size, 0.4 mm). During treatment planning, a single moving arc spanning 130 degrees was centred on the tumour using CT guidance and the motorised beam collimator size was conformed to the tumour volume (between 7-10mm in both axes). Dose was calculated on the planning software using a superposition–convolution dose engine (1, 2) and delivered on the SARRP using treatment mode at a dose rate of $\approx$ 2.5 Gy/min (peak energy, 225 kVp; beam filtration, 0.15 mm Cu; maximum tube current, 13.0 mA; focal spot size, 3.0 mm) . Dose distributions were calculated on heterogeneous electron densities obtained from manual intensity thresholding on CBCT, assuming water density for all soft tissues, bone density for bones, and air for regions outside the animal. Source-to-axis distance (centre of the tumour) was fixed and bed positioning and total duration of X-ray delivery were adjusted based on dose calculation from the treatment planning software.

Supplementary Table 2. Differences between preclinical image-guided irradiators and conventional clinical linacs, and between dosimetric characteristics of output ionising photon radiation. Note: MFP, electron mean free path; D_{max}, maximal dose in percent depth dose graphs; LET, linear energy transfer; Z, atomic number; CT, computed tomography; PTV, planned target volume; OAR, organ at risk.

	**Preclinical RT**	**Clinical RT**
**Tube acceleration potential (maximum photon energy)**	220 kV (220 keV)	6 MV (6 MeV) or 10 MV (10 MeV)
**Skin/surface relative dose**	High (about 100% of D_{max} at skin)	Low (about 40% of D_{max} at skin)
**Dose relative fall-off**	Fast (about 40% of D_{max} at 5 cm depth)	Slow (about 80% of D_{max} at 5 cm depth)
**Secondary electrons range**	Short (MFP $\approx$ 1-2 mm)	Long (MFP $\approx$ 10-20 mm)
**Linear energy transfer (LET)**	Intermediate (LET $\approx$ 3-4 keV/µm)	Low (LET $\approx$ 0.2 keV/µm)
**Dominant photon interaction**	*Photoelectric Effect*<br>-All photon energy is transferred in medium and electrons are emitted<br>-Energies <50 keV<br>-Mass absorption coefficient proportional to Z^3<br>-Strong backscattering	*Compton Scattering*<br>-Photon transfers some energy to an outer shell electron which is ejected, and then scatters<br>-Energies in 100 keV-10 MeV range<br>-Mass absorption coefficient independent of Z<br>-Forward directed
**Imaging modality for treatment planning**	*Cone-beam CT*<br>-X-ray tube voltage = 60 kVp<br>-X-ray tube current = 13 mA<br>-Low contrast, prone to image artefacts and fixed resolution<br>-Cone-beam multi-slice simultaneous tomographic acquisition	*CT simulator*<br>-X-ray tube voltage = 70-140 kVp<br>-X-ray tube current = 50-800 mA with automated exposure control<br>-Reconstruction kernel adapted to organ site and desired contrast/resolution<br>-Slice-by-slice tomographic acquisition
**Target delineation for treatment planning**	Tumour is targeted with or without PTV definition (not enough contrast to segment)	PTV and OARs are segmented and plan is optimised to limit dose to OARs

B. Radiobiological comparison of HFRT (5 × 5 Gy) and SDRT (1 × 20 Gy). In the main study we compared a hypofractionated regimen (HFRT, 5 × 5 Gy = 25 Gy) with an ablative single fraction regimen (SDRT, 1 × 20 Gy = 20 Gy) delivered on the SARRP. In clinical RT, total dose is critical and here we show that, once a high–dose-per-fraction formalism is used with the generalised linear-quadratic (gLQ) model (3), the two regimens have similar biological effectiveness and can reasonably be compared in the context of our imaging study.

Using the conventional linear-quadratic (LQ) model, for n fractions of dose d and a tissue with α/β, we can define the biologically effective dose (BED) and the equivalent dose in 2 Gy fractions (EQD2)

$$\mathrm{BED} = nd\left(1+\frac{d}{\alpha/\beta}\right), \qquad \mathrm{EQD2} = \frac{\mathrm{BED}}{1+\frac{2}{\alpha/\beta}}.$$

With $\alpha/\beta = 4$ Gy typically used for breast cancers (4):

- HFRT, $n = 5$, $d = 5$ Gy:

$$\mathrm{BED}_{\mathrm{HFRT}} = 5\times 5\left(1+\frac{5}{4}\right) = 25\times 2.25 = 56.25\ \mathrm{Gy},$$

$$\mathrm{EQD2}_{\mathrm{HFRT}} = \frac{56.25}{1+\frac{2}{4}} = \frac{56.25}{1.5} = 37.5\ \mathrm{Gy}.$$

- SDRT, $n = 1$, $d = 20$ Gy:

$$\mathrm{BED}_{\mathrm{SDRT}} = 20\left(1+\frac{20}{4}\right) = 20\times 6 = 120\ \mathrm{Gy},$$

$$\mathrm{EQD2}_{\mathrm{SDRT}} = \frac{120}{1.5} = 80\ \mathrm{Gy}.$$

The conventional LQ model therefore makes the single 20 Gy fraction look much more effective than the hypofractionated 5 × 5 Gy regimen. This is exactly the known limitation of the standard LQ model when used at high dose per fraction. Indeed, at doses >8–10 Gy per fraction, experimental survival curves often deviate from the purely quadratic behaviour assumed by the LQ model, because sublethal damage can be repaired during delivery and because the curve straightens. Wang *et al.* proposed the gLQ model that reduces to standard LQ at conventional doses and dose rates, incorporates the actual delivery time and a repair constant, and gives more realistic estimates for stereotactic/ablative regimens, such as the SDRT scheme used in our study (3).

For completeness, we define below parameters used in Wang *et al.*. Considering a single fraction of total dose D delivered at a constant dose rate I_0 over time T:

$$D = I_0 T.$$

Let m be the first-order repair constant

$$m = \frac{\ln 2}{T_{1/2}},$$

where $T_{1/2}$ is the repair half-time (we use $T_{1/2} = 30$ min, i.e. $m \approx 0.0231\ \mathrm{min}^{-1}$). Let α and β be the usual LQ parameters, and let

$$b_2 = \sqrt{\beta}$$

and the repair factor e,

$$e = m + b_2 I_0,$$

and the time factor G,

$$G = \frac{2}{(eT)^2}\left(eT - 1 + e^{-eT}\right).$$

Then the log-cell kill for that fraction, or its effect, E, is defined as

$$E = \alpha D + \beta G D^2.$$

For multiple fractions $i = 1, \ldots, n$ delivered on different days:

$$E_{\text{tot}} = \sum_{i=1}^{n} \left(\alpha d_i + \beta G_i d_i^2\right),$$

with $d_i = I_0 T_i$ and G_i computed as above for each fraction. Finally, to express E_{tot} as an EQD2 we divide by the effect per 2 Gy fraction:

$$\text{EQD2} = \frac{E_{\text{tot}}}{\alpha + 2\beta}.$$

We used the SARRP delivery conditions with dose rate $I_0 = 2.5$ Gy/min, HFRT with 5×5 Gy result in time of $T = 5/2.5 = 2.0$ min per fraction, and SDRT with 1×20 Gy result in $T = 20/2.5 = 8.0$ min. For breast cancer xenografts (4), it is reasonable to assume

$$\alpha = 0.30 \text{ Gy}^{-1}, \qquad \alpha/\beta = 4 \text{ Gy} \quad \Rightarrow \quad \beta = \frac{\alpha}{\alpha/\beta} = \frac{0.30}{4} = 0.075 \text{ Gy}^{-2}.$$

We assume a repair half-time of

$$T_{1/2} = 30 \text{ min} \quad \Rightarrow \quad m = \frac{\ln 2}{T_{1/2}} = \frac{\ln 2}{30} \approx 0.0231 \text{ min}^{-1}.$$

Following Wang *et al.* we set

$$b_2 = \sqrt{\beta} = \sqrt{0.075} \approx 0.274,$$

and compute

$$e = m + b_2 I_0 = 0.0231 + 0.274 \times 2.5 = 0.0231 + 0.685 \approx 0.708 \text{ min}^{-1}.$$

Hence for HFRT, the gLQ time factor G is

$$G = \frac{2}{(eT)^2}\left(eT - 1 + e^{-eT}\right) = \frac{2}{(1.416)^2}\left(1.416 - 1 + e^{-1.416}\right).$$

$$G \approx 0.657 \quad .$$

The effect per 5 Gy fraction is therefore

$$E_{\text{frac}} = \alpha d + \beta G d^2 = 0.30 \times 5 + 0.075 \times 0.657 \times 25.$$

$$E_{\text{frac}} = 1.50 + 1.875 \times 0.657 = 1.50 + 1.232 = 2.732.$$

For 5 fractions:

$$E_{\text{HFRT}} = 5 \times 2.732 = 13.66.$$

To express this as EQD2 we divide by the effect of a 2 Gy fraction, *i.e.*

$$\text{EQD2}_{\text{HFRT}} = \frac{E_{\text{HFRT}}}{\alpha + 2\beta} = \frac{13.66}{0.45} = 30.36 \text{ Gy} \approx 30.4 \text{ Gy}.$$

Thus, the HFRT course is biologically equivalent to about 30.4 Gy EQD2. For SDRT, with the same m and b_2 as above,

$$G = \frac{2}{(5.66)^2}\left(5.66 - 1 + e^{-5.66}\right) \approx 0.29.$$

Hence

$$E_{\mathrm{SDRT}} = \alpha D + \beta G D^2 = 0.30 \times 20 + 0.075 \times 0.29 \times 400,$$

$$E_{\mathrm{SDRT}} \approx 6.0 + 8.7 \approx 14.7,$$

and

$$\mathrm{EQD2}_{\mathrm{SDRT}} = \frac{14.7}{0.45} \approx 32.7 \text{ Gy}.$$

Using the gLQ model, we obtain:

$$\mathrm{EQD2}_{\mathrm{HFRT}} \approx 30.4 \text{ Gy}, \qquad \mathrm{EQD2}_{\mathrm{SDRT}} \approx 32.7 \text{ Gy}.$$

The SDRT scheme is only around one additional 2 Gy fraction more effective in EQD2 terms than the HFRT scheme, making them biologically similar. Overall, the physical doses (25 vs 20 Gy) look different, but their biological effectiveness is similar once a high-dose model with real delivery times is employed. Both regimens are equivalent to $\sim$ 15-16 fractions of 2 Gy, so comparing these schemes is reasonable.

C. Mesoscopic photoacoustic image acquisition and analysis. Mesoscopic photoacoustic imaging (PAI) raster-scanning acquisitions were conducted using a commercial system (RSOM; Explorer P50, iThera Medical GmbH) in a 12 x 12 mm^2 field of view at 20 µm step size, with 3 ns-long laser pulses of 80 mJ in energy at 1 kHz pulse repetition rate, synchronised with ultrasound detection using a single-element transducer with a centre frequency of 50 MHz ($\approx 90\%$ bandwidth). Total scan time for mesoscopic PAI was ≈7 min.

Motion corrected mesoscopic PAI were reconstructed in 3D using a beam-forming algorithm (5, 6), with in-plane resolution 20 × 20 µm^2 and axial resolution of 4 µm. Hand-drawn regions of interest (ROI) were selected on Z-slices throughout the reconstructed segmented 3D images by an experienced user (LCW), avoiding skin vessels and artefacts, using intensity images for quality control verifications. All tumour ROIs were validated by an expert user (TLL). Mesoscopic PAI metrics summary is provided in Supplementary Table 3.

D. Tomographic multispectral photoacoustic image acquisition and analysis. Prior to tomographic PAI acquisition using a commercial system (MSOT; inVision, iThera Medical GmbH), anaesthetised mice were positioned, stabilised and acclimatised in the system's water bath maintained at 36°C for 10 min. Tomographic acquisitions were performed on the largest tumour cross-section with 6 averages per wavelength (700, 730, 750, 760, 770, 800, 820, 840, 850, and 880 nm). The total scan time for tomographic PAI including the gas challenge was ≈20 min.

Using the Python photoacoustic tomography analysis toolkit (PATATO) (7), averaged PAI tomographic images were reconstructed using a back-projection algorithm with in-plane resolution of 75 x 75 µm^2 at each acquired wavelength. Spectral image series were then downsampled to a pixel size of 225 x 225 µm^2 to increase the signal-to-noise ratio and pixel-wise unmixing was conducted across wavelengths using oxy- and deoxy-haemoglobin absorption spectra. Tumour ROIs were manually selected on the largest tumour cross-section by two experienced users (LCW, TLL). A small ROI around a portion of the large femoral artery was also captured to identify changes in blood oxygenation as a reference. All parametric maps were quantified in manually delineated tumour and reference ROIs. We make a note that PAI-derived sO_2 reported throughout this manuscript is an estimate and not a direct interrogation of true/absolute blood oxygen saturation. Gas challenge tomographic data was analysed using sO_2 time-series data. For assessing how sO_2 changed after switching the breathing gas, the time-point of change to pure oxygen was identified in a reference region selected in the large femoral artery on tomographic PAI. Hence, we quantified and reported the change in sO_2 (ΔsO_2) under gas challenge in the tumour area by taking the percent change between averaged time-series data before and after that time-point (Supplementary Figure 5). Responding fraction (RF) was assessed as the ratio of voxels with ΔsO_2 greater than one standard deviation above the baseline mean sO_2 before gas change. Tomographic PAI metrics summary is provided in Supplementary Table 3.

Supplementary Table 3. Summary table of extracted quantitative photoacoustic imaging biomarkers in both modalities. Note: i, pixel element; N, total pixel within region of interest.

Modality	Quantitative imaging biomarker	Physical quantity measured	Biological processes represented
Multispectral tomographic photoacoustic imaging	Total haemoglobin (THb), sum of deoxy-haemoglobin (HbR) and oxyhaemoglobin (HbO_2)	THb = $\sum_{i=1}^{N}(HbR_i + HbO_{2i})/N$	Blood content
	Blood oxygen saturation (sO_2)	$sO_2 = \sum_{i=1}^{N}(HbO_{2i} / (HbR_i + HbO_{2i}))/N$	Tissue blood oxygenation
	Standard deviation (SD) of sO_2	SD of $sO_2 = \sqrt{\sum_{i=1}^{N}(sO_{2i}-sO_2)^2/N}$	Intratumoural blood oxygen heterogeneity
	Change in sO_2 under gas challenge (ΔsO_2) and responding fraction (RF)	$\Delta sO_2 = sO_2^{100\% O_2} - sO_2^{Air}$; RF defined as pixel count with ΔsO_2 >mean + 1 SD of ΔsO_2 over total number of pixels	Tissue oxygen diffusion
Monospectral mesoscopic photoacoustic imaging	Blood volume (BV)	Total pixels in segmented vasculature	Superficial perfused tumour blood content
	Diameter	Average segments diameter in segmented skeletonised vascular network	Diameter of vessels across the tumour periphery
	Loop 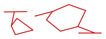	Count of groups of vertices connected by path forming a closed structure in segmented skeletonised vascular network	Looping or curving structures in the vasculature at the tumour periphery
	Connected component (CC) 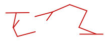	Count of groups of vertices connected by path with edges in segmented skeletonised vascular network	Connectivity of the vasculature
	Density	Counts of vessel segments normalised to BV	Vessel coverage in tumour periphery

E. Immunohistochemistry image analysis. Analyses were conducted within the whole viable tumour area, excluding skin and necrotic regions, identified on H&E. In HALO (v3.2, Indica Labs), random-forest classifiers were trained by an expert user (CB) for distinguishing between the necrotic and viable tissue regions. Classifiers were trained using at least 50 annotations per tissue class per image on a training set of 20-40% of the images and created independently for each stain and then applied across the scanned stained sections. Positive areas of CD31 and ASMA were quantified as a percentage of the total classified tumour area using the area quantification module (v2.4.3). The percentage of positive cells within the classified tumour regions were quantified for Ki67, γ-H2AX, and HIF1-α with the multiplex IHC module (v3.1.4). To quantify ASMA coverage of CD31-positive tumour vasculature, the CD31 random forest classifier was overlaid onto the ASMA section and its classifier, taking the intersection of the areas as the ASMA vessel coverage, and was reported as a percent of the total viable tumour area (Supplementary Figure 6).

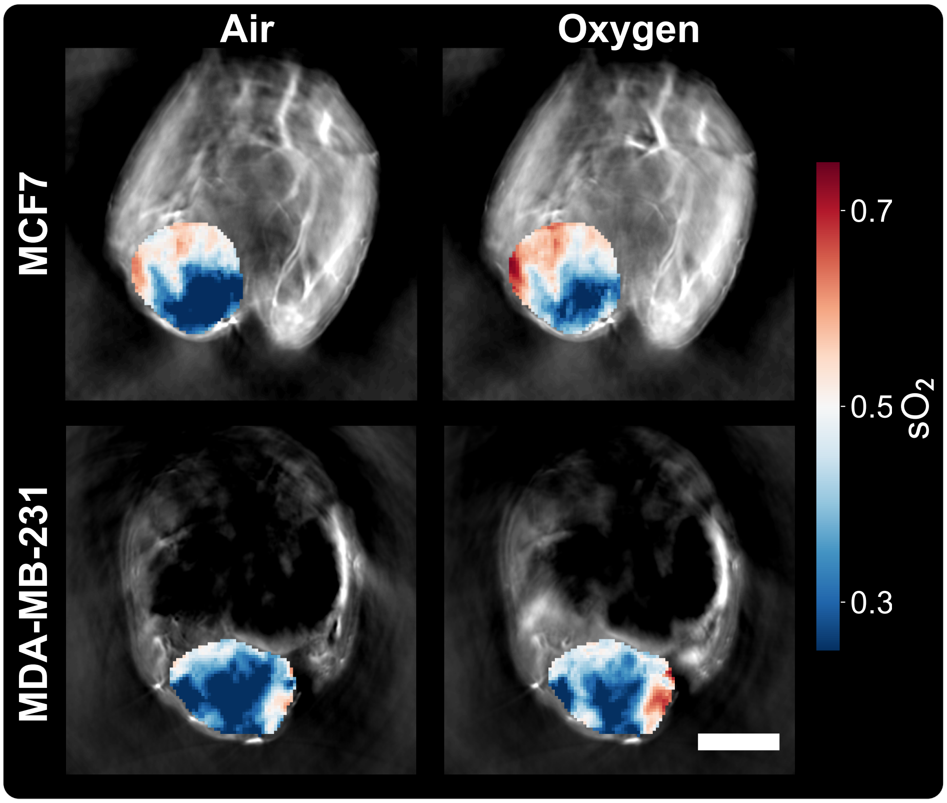

Supplementary Figure 5. Exemplar MCF7 and MDA-MB-231 tumour-bearing mice at baseline imaged with multispectral tomographic PAI with quantitative parametric maps of blood oxygen saturation (sO_2) under gas challenge. Tumour sO_2 displays higher deoxyhaemoglobin content (blue) under breathing air (20% O_2), and higher oxyhaemoglobin content (red) when breathing gas is switched to pure oxygen delivery (100% O_2). Calculation of the pixel-wise difference between the two parametric maps allow the quantification of the change in sO_2 and the responding fraction. Scale bar, 2 mm

Supplementary Table 4. Immunohistochemistry and histopathological markers used for staining processed tumour sections and quantification.

Antibody	Supplier	Retrieval method	Visualisation	Quantification
Anti-mouse Cluster of Differentiation 31 (CD31)	Cell Signaling, 77699	1:100, Tris-EDTA HIER 20min	Stable and highly expressed endothelial cell marker	Percent CD31 positive area in non-necrotic viable tumour classified with trained random forest classifier
Anti-mouse Alpha Smooth Muscle Actin (ASMA)	Abcam, ab5694	1:500, Tris-EDTA HIER 10min	Smooth muscle and pericyte marker; indicates vascular maturity	Percent ASMA positive area in non-necrotic viable tumour classified with trained random forest classifier; overlaid with CD31-classified area for capturing double CD31-ASMA area
Anti-human Ki67	Daco, Agilent, M7240	1:400, Tris-EDTA HIER 30min	Protein expressed in active phases of cellular division, marker of proliferation	Percent Ki67 positive nuclei over all nuclei in non-necrotic viable tumour classified with trained random forest classifier
Anti-human phosphorylated H2A Histone Family Member X (γ-H2AX)	Cell Signaling, 9718	1:200, Sodium Citrate HIER 20min	Core protein that structures DNA into chromatine and becomes phosphorylated with strand breaks, marker of DNA damage	Percent γ-H2AX positive nuclei over all nuclei in non-necrotic viable tumour classified with trained random forest classifier
Anti-human Hypoxia inducible factor 1-α (HIF1-α)	Abcam, ab51608	23.36 µg/ml, Sodium Citrate HIER 20min	Acute hypoxia marker	Percent HIF1-α positive nuclei over all nuclei in non-necrotic viable tumour classified with trained random forest classifier
Haematoxylin & Eosin (H&E)	Leica Microsystems Eosin 1%, Haem. 3801560E	–	Combined nuclear and cytoplasm/extracellular tissue markers	Percent necrotic area classified across the whole tumour, excluding skin regions for all analyses, with trained random forest classifier

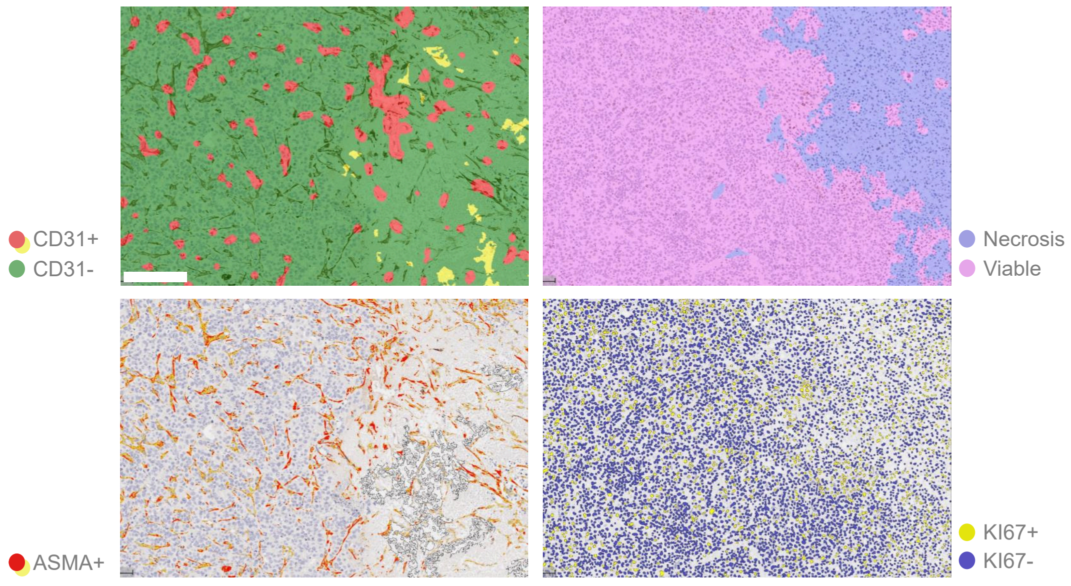

Supplementary Figure 6. Immunohistochemistry (IHC) analysis of resected tumour tissue sections from breast cancer xenograft. ASMA stained IHC panel (left) with overlaid CD31 classifier (top) trained using HALO showing areas classified as positive (red) and negative (green), and with overlaid ASMA classifier (bottom) showing positive areas (strongly positive in red). Ki67 sequential stained IHC section panel with overlaid necrosis (purple) vs. viable tissue (pink) classifier (top) and with overlaid Ki67 positive nuclei (yellow) and negative nuclei (blue).

Supplementary Note 3: Enabling clinically relevant preclinical image-guided radiation therapy through dosimetric quality assurance

Prior to conducting any *in vivo* experiments, dosimetric quality assurance (QA) of the small animal radiation research platform (SARRP, Xstrahl) was performed to ensure that the planned dose corresponded to the actual delivered dose in tissue-equivalent phantoms (solid-water mass density (g/cm^3), 1.032 ± 0.005; electron density (e^-/cm^3), 0.557 ± 0.001; Sun Nuclear). Dose verification was performed with a National Physical Laboratory-calibrated ionisation chamber (Farmer, PTW Dosimetry) in standard solid-water phantoms in reference conditions (Supplementary Figure 7). The American Association of Physicists in Medicine (AAPM) protocol for 40–300 kV X-ray beam dosimetry was followed (8), using AAPM task group 51 formalism and the measured absolute dose was compared to the reference dose rate of 3.28 Gy/min measured at the initial SARRP commissioning (Supplementary Table 5). It is recommended that differences between prescribed dose and delivered dose be 5% or less (9). Hence, each of the steps including treatment planning should introduce uncertainties under 5% and should add up to a maximum of 5%. In preclinical RT, even though we aim to reduce errors as low as reasonably achievable, higher uncertainties are typically tolerated, with maximal dose output differences of 10% (10). We also point the reader back to the differences to consider between preclinical and clinical RT summarised in Supplementary Table 2.

Finally, prior to treatment, fixed beam delivery was compared to moving arc delivery after manually segmenting tumours and surrounding tissue or organs at risk (OAR) as two distinct instances on CBCT images, and resulting dose-volume histograms were assessed (Supplementary Figures 8 and 9). This testing was conducted considering that preclinical irradiators are typically used solely in a top-irradiation fixed set-up. With the improved capabilities of image-guided irradiators, we were provided with the ability to give more conformal treatment and briefly attempted to show the added value of using the SARRP's abilities through image guidance and moving arc delivery. The implementation of a QA protocol confirmed the suitability of our preclinical RT framework described in the next subsection.

Absolute dosimetry in reference conditions (Supplementary Figure 7) showed that the dose output factor of the SARRP was within 3.0% of the reference dose at the SARRP's commissioning across three measurement time-points over the years of the study conduction (min-max measured dose error, 1.2-3.0 %; Supplementary Table 5).

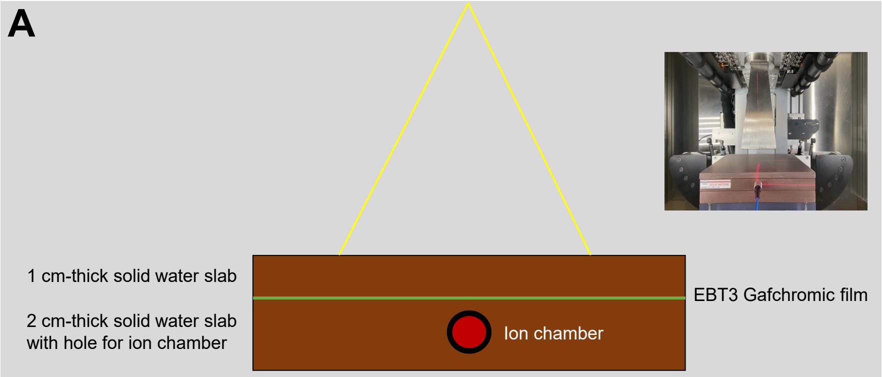

Supplementary Figure 7. Experimental setup for the dosimetric survey of the SARRP. A) Dosimetry in reference standard conditions. Solid water phantoms are positioned with fixed source-to-axis distance, radiation beam is turned on for a given amount of time, and dose is measured with an ionisation chamber within the phantom insert by collecting charges which amount is corrected by assessing standard correction factors in different irradiation setups.

Supplementary Table 5. Absolute dosimetry of the Small Animal Radiation Research Platform. Raw charge measurements, corrected charge measurements and resulting dose compared to calibration.

	Repeat 1	Repeat 2	Repeat 3	Reference
**Date**	19/11/2020	27/01/2021	19/02/2025	
**Exposure time (s) [Repeats]**	60 [18]	60 [12]	60 [12]	
**Raw collected charges (nC)**	64.61 $\pm$ 0.03	57.59 $\pm$ 0.012	56.64 $\pm$ 0.03	
**Measured correction factors**	1.002 $\pm$ 0.002	1.031 $\pm$ 0.002	0.992 $\pm$ 0.003	
**Corrected collected charges (nC)**	64.74	59.94	56.06	
**Dose output (Gy)**	3.36	3.09	2.92	3.28
**Error (%)**	1.2	3.0	5.9	

Comparing fixed vs. moving arc delivery, the resulting isodose lines with moving arc delivery spanning 135°were more conformal to the tumour and less dose was delivered to surrounding organs at risk (OAR) (Supplementary Figure 8). The 80% isodose line systematically went deeper within healthy organs with the fixed beam delivery, further illustrated in the dose-volume histograms (DVH, Supplementary Figure 9). Fixed beam and moving arc deliveries achieved similar dose distribution, with around 80% of the segmented tumour receiving 100% of the dose, either 5.0 Gy in a one fraction delivery of the HFRT scheme or 20.0 Gy in the SDRT scheme. However, for OARs sparing, 7-8% more of the OAR volume received 50% of the dose with fixed beam than with moving arc delivery, equating to 2.5 Gy for HFRT or 10.0 Gy for SDRT (48% or 57% of OAR volume with fixed beam vs. 23% or 31% of OAR volume with moving arc, respectively; see arrows in Supplementary Figure 9). More alarming was that 21-23% more of OAR volume received 75% of the planned dose when using fixed beam, that is 3.75 or 15.0 Gy (44% or 54% of OAR volume with fixed beam vs. 40% or 50% of OAR volume with moving arc, respectively; see arrows in Supplementary Figure 9). Dose to the skin was always higher than the planned dose (105%, Supplementary Figure 8), which is likely a direct result of the strong backscattering component of 220 kVp beams (Supplementary Table 2). Electrons produced by photoelectric interaction in the tissue are mostly backward-directed and deposit the dose at shallow depths (mean e^- free path = 1-2 mm). Better dose homogeneity was achieved and the higher skin dose was more conformed to the tumour skin rather than to surrounding OAR skin in moving arc plans. Thus, moving arcs were employed for all subsequent *in vivo* RT experiments performed in this study, with the confidence that the delivered dose was within reasonable tolerance threshold (<5%).

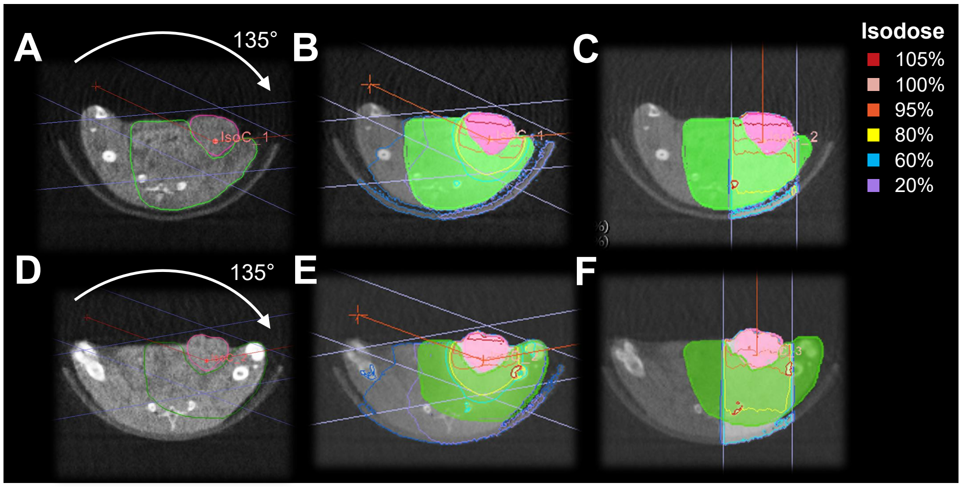

Supplementary Figure 8. Treatment planning on CBCT axial slices in A,D) two mice treated with moving arcs. Dose distributions with isodose lines overlaid on CBCT and filled segmentations of the PTV (pink) and of surrounding OAR (green) for two treatment scenarios with the B,E) moving arcs and C,F) fixed beam. Isodose lines represent percent of planned dose to PTV.

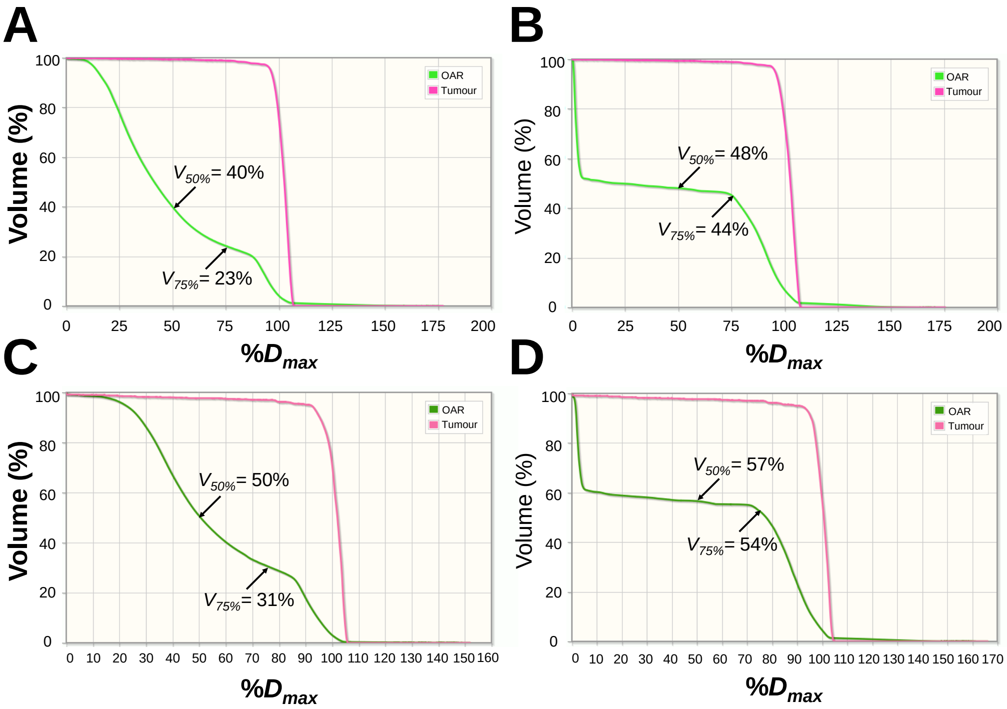

Supplementary Figure 9. Dose-volume histograms (DVH) for two treatment scenarios in two breast cancer xenografts with the A,C) moving arcs or B,D) fixed beam. Dosimetric characteristics of target volumes (pink) and organs at risk (green) treated depict that both moving arcs and fixed beam achieved similar target coverage. The former achieved better organs at risk sparing with a mean healthy volume receiving 75% of the dose lower in the two exemplar mice.

Supplementary References

1. Robert Jacques, Russell Taylor, John Wong, and Todd McNutt. Towards real-time radiation therapy: Gpu accelerated superposition/convolution. *Computer Methods and Programs in Biomedicine*, 98(3):285–292, 2010.
2. Robert Jacques, John Wong, Russell Taylor, and Todd McNutt. Real-time dose computation: Gpu-accelerated source modeling and superposition/convolution. *Medical Physics*, 38(1):294–305, 2011.
3. Jian Z. Wang, Zhibin Huang, Simon S. Lo, William T. C. Yuh, and Nina A. Mayr. A generalized linear-quadratic model for radiosurgery, stereotactic body radiation therapy, and high–dose rate brachytherapy. *Science Translational Medicine*, 2(39):39ra48–39ra48, 2010.
4. C. M. van Leeuwen, A. L. Oei, J. Crezee, A. Bel, N. A. P. Franken, L. J. A. Stalpers, and H. P. Kok. The alfa and beta of tumours: a review of parameters of the linear-quadratic model, derived from clinical radiotherapy studies. *Radiation Oncology*, 13(1), 2018.
5. S. Park, A. B. Karpiouk, S. R. Aglyamov, and S. Y. Emelianov. Adaptive beamforming for photoacoustic imaging. *Opt Lett*, 33(12):1291–3, 2008.
6. M. Omar, D. Soliman, J. Gateau, and V. Ntziachristos. Ultrawideband reflection-mode optoacoustic mesoscopy. *Opt Lett*, 39(13):3911–4, 2014.
7. Thomas R. Else, Janek Grohl, Lina Hacker, and Sarah E. Bohndiek. Patato: a python photoacoustic tomography analysis toolkit. *Journal of Open Source Software*, 9(93):5686, 2024.
8. C. M. Ma, C. W. Coffey, L. A. DeWerd, C. Liu, R. Nath, S. M. Seltzer, J. P. Seuntjens, and Medicine American Association of Physicists in. Aapm protocol for 40-300 kv x-ray beam dosimetry in radiotherapy and radiobiology. *Med Phys*, 28(6):868–93, 2001.
9. B. Fraass, K. Doppke, M. Hunt, G. Kutcher, G. Starkschall, R. Stern, and J. Van Dyke. American association of physicists in medicine radiation therapy committee task group 53: quality assurance for clinical radiotherapy treatment planning. *Med Phys*, 25(10):1773–829, 1998.
10. P. E. Lindsay, P. V. Granton, A. Gasparini, S. Jelveh, R. Clarkson, S. van Hoof, J. Hermans, J. Kaas, F. Wittkamper, J. J. Sonke, F. Verhaegen, and D. A. Jaffray. Multi-institutional dosimetric and geometric commissioning of image-guided small animal irradiators. *Med Phys*, 41(3):031714, 2014.
